# Reactive anti-predator behavioral strategy shaped by predator characteristics

**DOI:** 10.1371/journal.pone.0256147

**Published:** 2021-08-18

**Authors:** Meredith S. Palmer, Craig Packer

**Affiliations:** Department of Ecology, Evolution, and Behavior, University of Minnesota, St. Paul, Minnesota, United States of America; Sichuan University, CHINA

## Abstract

Large mammalian herbivores use a diverse array of strategies to survive predator encounters including flight, grouping, vigilance, warning signals, and fitness indicators. While anti-predator strategies appear to be driven by specific predator traits, no prior studies have rigorously evaluated whether predator hunting characteristics predict reactive anti-predator responses. We experimentally investigated behavioral decisions made by free-ranging impala, wildebeest, and zebra during encounters with model predators with different functional traits. We hypothesized that the choice of response would be driven by a predator’s hunting style (i.e., ambush vs. coursing) while the intensity at which the behavior was performed would correlate with predator traits that contribute to the prey’s relative risk (i.e., each predator’s prey preference, prey-specific capture success, and local predator density). We found that the choice and intensity of anti-predator behaviors were both shaped by hunting style and relative risk factors. All prey species directed longer periods of vigilance towards predators with higher capture success. The decision to flee was the only behavior choice driven by predator characteristics (capture success and hunting style) while intensity of vigilance, frequency of alarm-calling, and flight latency were modulated based on predator hunting strategy and relative risk level. Impala regulated only the intensity of their behaviors, while zebra and wildebeest changed both type and intensity of response based on predator traits. Zebra and impala reacted to multiple components of predation threat, while wildebeest responded solely to capture success. Overall, our findings suggest that certain behaviors potentially facilitate survival under specific contexts and that prey responses may reflect the perceived level of predation risk, suggesting that adaptive functions to reactive anti-predator behaviors may reflect potential trade-offs to their use. The strong influence of prey species identity and social and environmental context suggest that these factors may interact with predator traits to determine the optimal response to immediate predation threat.

## Introduction

When encountering a predator, prey can perform a number of different behaviors to survive or mitigate a possible attack [1, 2]. These responses can broadly be categorized as defensive behaviors, visual or auditory signaling, enhanced vigilance or monitoring, and evasive actions. Different behavioral categories are hypothesized to have contrasting functions (Table 1), such as alerting conspecifics to imminent risk [3, 4], informing predators that they have been detected [5, 6], signaling high levels of physical fitness [1, 2, 7], monitoring or detecting additional predators [8, 9], diluting individual predation risk [10–12], or fleeing the immediate area [13, 14]. As predators vary in key traits which may render particular responses more or less effective [15–17], the anti-predator behaviors performed may be chosen based on the specific threat faced [13, 18, 19]. However, we still know little about which elements of predatory threat shape variation in reactive anti-predator strategies in large mammals [1, 2].

**Table 1:** Reactive anti-predator behaviors performed by large terrestrial mammals when encountering predators, their hypothesized adaptive functions (adapted from [20]), and predictions of the most effective response to each hunting style.

Category of response	Types of behavior	Hypothesized function	Predicted effectiveness against
Defense	Clumping/huddling	Warding off or resisting predator attack	Ambush hunters
	Group attack/mobbing		
Alerting	Snorting, barking, whistling	Conveying information to conspecifics or predator that the predator has been detected	Ambush hunters
	Tail-flagging		
	Foot-stamping		
Vigilance	Vigilance (general/low intensity)	Detecting, monitoring predator; assessing predation threat	Ambush hunters
	Vigilance (specific/high intensity)		
	Predator inspection		
Evasion	Seeking refuge	Evacuating the immediate area or hiding from detection	Coursing hunters
	Freezing		
	Flight		
Fitness indicators	Stotting/pronking (exaggerated leaps)	Conveying information to predators that prey is so vigorous as to be unlikely to be caught	Coursing hunters
	Tacking/zig-zagging (exaggerated running)		

Hunting style in terrestrial predators ranges along a gradient from ambush hunting to active pursuit. Predators on the ambush end of the spectrum depend on surprise and a quick chase to bring down prey while coursing predators rely more on stamina to capture prey after long-distance pursuits [21]. Empirical studies in vertebrates indicate that certain categories of behavior are more commonly used during encounters with particular hunting types, suggesting these behaviors may increase chances of surviving the predation attempt. A prime example is Thomson’s gazelle (*Eudorcas thomsonii*), which employ stotting and other honest signals of fitness when facing coursing predators but react to ambush predators using alarm cues and predator inspection [7, 22–24]. If particular behaviors function to improve escape probability from predators with different hunting styles, we would expect that similar categories of response would be performed across prey species. While invertebrate prey predictably perform different suites of anti-predator behavior relative to predator hunting style [25, 26], little research has systematically evaluated the reactive responses of different vertebrate prey species to ambush versus pursuit predators.

Anti-predator decisions may also depend on the traits that contribute to the perceived level of risk posed by each predatory species [27, 28]. Overall predation risk can be characterized by the combined likelihoods of each stage in the predation process, i.e., (i) encountering a predator, (ii) being attacked given an encounter, and (iii) being killed given an attack [13, 17, 29]. Encounter probability reflects contact rates between prey and predators, which is a function of predator density (among other factors). When a prey animal has been discovered, a predator then chooses whether to attack. This may depend in part on predator preference for specific prey, with some species being selected or avoided relative to overall availability [30–33]. If a hunt is initiated, the likelihood that it will end in prey capture is typically low (large terrestrial predators rarely average hunting success rates of >50%) and varies by prey [34, 35]. There is some evidence that mammalian prey increase the intensity of anti-predator behavioral performance when confronting more dangerous predators: black-tailed deer (*Odocoilus hemionus sitkensis*) feed less in areas treated with urine of more lethal predators (stage iii; [28, 36] and zebra (*Equus quagga*) and Thomson’s gazelle maintain greater distances from predators that select for them more heavily (stage ii [21, 22]). The frequency, duration, or swiftness to perform certain behaviors in encounters with specific predators may therefore reflect relative risk, but we lack systematic studies that tease apart how each element of the predation process drives the performance of anti-predator behaviors [17, 29].

**Fig 1:**
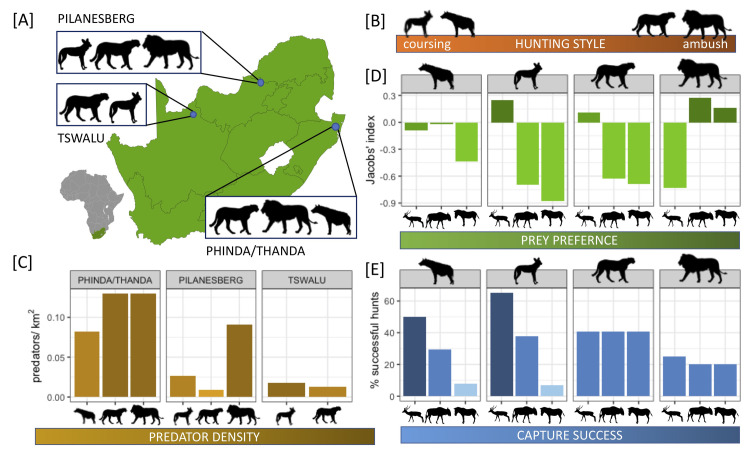
**[A] Map** of study sites with [B-E] characteristics of spotted hyenas, wild dogs, cheetahs, and lions which may influence prey’s choice of anti-predator behavioral response. **[B] Hunting style**, dichotomized as coursing or ambush; **[C] Predator density**, in predators/km^2^**; [D] Prey preference**, the degree to which predators select for each individual prey species, represented by a Jacobs’ preference index which ranges from -1 (strong avoidance) to +1 (strong preference); **[E] Capture success**, the proportion of initiated hunts that end in a kill for each prey species. See text for references.

Here, we conduct a rigorous experiment to generate a framework for predicting anti-predator strategies based on predator traits. We used life-sized, mobile models to elicit behavioral responses in three large African herbivore species (impala, wildebeest, and zebra) towards four carnivores (lion *Panthera leo*, cheetah *Acinonyx jubatus*, wild dog *Lycaon pictus*, and spotted hyena *Crocuta crocuta* with impala and Thomson’s gazelle controls) that differed in hunting style and prey species-specific threat level. We predicted that:

- (H1) the *type* of behavioral response chosen would be dictated by *predator hunting style*, with all prey species directing alerting, defensive, and vigilance behaviors towards predators that utilize surprise for capture success (ambush hunters), while using immediate flight and indicators of good health in response to predators that rely on endurance to capture prey (coursing hunters) (Table 1). As not all focal species are morphologically capable of performing the same actions (e.g., zebra cannot stot), we focused on the category of response rather than specific behaviors.
- (H2) the *intensity* of anti-predator response would reflect the *relative risk posed* by each predator, with prey displaying more numerous, longer, or swifter responses towards predators that were frequently encountered, more likely to attack, or highly successful at hunting that particular prey species [21, 28, 36] (Fig 1).

## Materials and methods

### Study sites

Experiments were performed in three South African wildlife areas during Jun-Aug 2015 and Jun-Oct 2016 (Fig 1A, S1 Table). (1) Phinda Private Game Reserve (210 km^2^; 27°50’S, 32°26’E) and Thanda Private Game Reserve (50 km^2^; 27°52’S, 32°10’E) are in the Maputaland region of KwaZulu-Natal. Situated within 10 km of each other inside a contiguous corridor of fenced reserves, these sites contain equivalent predator and prey communities and vegetation types (lowveld, bushveld, and grassland [37]). As such, we grouped experiments from both locations into a single category, while using the predator densities specific to each individual reserve for our analyses. The Phinda/Thanda complex contains lion, hyena, and cheetah. (2) Tswalu Kalahari Reserve (1,000 km^2^; 27°13’S, 22°28’E) is located in the Northern Cape region and is comprised of dune, bushveld, grassland, and savanna habitat [38]. We worked within an 800 km^2^ fenced section of the reserve containing cheetah and wild dog. (3) Pilanesberg National Park (580 km^2^; 25°15’S, 26°57’E) lies within the North West Province and includes bushveld, grassland, and savanna habitats [39]. The Pilanesberg predator community contains lion, cheetah, and wild dog. As these sites differed in carnivore community composition, we did not present predator models to prey lacking ecological experience with the missing species.

### Focal prey

We focused on three large African herbivores that were abundant across study sites: impala, blue wildebeest, and plains zebra. These species are consumed by all four predators, but with varying degrees of susceptibility and selection.

### Predator characteristics

We note that factors such as prey age-sex class, predator hunting-group size, visibility, time of day, and other social and environmental factors may influence predator capture success, style, and preference and that behaviors such as hunting style lie along a spectrum [34, 35, 40–42]. We rely on expert knowledge and published literature to classify predator behaviors and consider the following values to represent relative indices or general trends of behavior rather than absolute metrics. Wherever possible, we used behaviors of predators recorded under conditions most similar to our study sites.

#### Hunting style

Lions and cheetah stalk and ambush their prey, relying on cover to closely approach prey before giving a short chase (“ambush hunters” [21, 35]). Wild dogs and hyena are “coursing” hunters, using endurance and long chases (sometimes up to several kilometers) to bring down prey [41, 43–45] (Fig 1B).

#### Predator density

We calculated the densities for each predator species in each reserve during our study ([46, 47], C. Pickering, pers. comm.). As predator densities varied across sites, these values were then scaled relative to the densities of the remaining focal predator species within each reserve to generate relative density estimates for each reserve (Fig 1C).

#### Prey preference

The degree to which each prey species was selectively hunted by each predator was taken from meta-analyses that evaluated predator selectivity across multiple countries where the three prey species coexist [30–33, 48] (Fig 1D). Relative selectivity was calculated using a Jacobs’ index, a value ranging from -1 (strong avoidance) to +1 (strong preference). The index accounts for the community and abundance of available prey species at each site and is not biased by predator hunting group size or sex ratios (see [30–33, 48] for further details).

#### Capture success

Capture success was defined as the percentage of initiated hunts that ended in a kill. Specific estimates for each predator-prey dyad were gathered from the literature, with preference given to those calculated at locations which were geographically proximate to our study areas [42, 49–51] (Fig 1E). We were unable to locate South African studies for hyena and used instead data from Kenyan populations [42]. Only for cheetah were we unable to find capture success values disaggregated by prey species and instead used overall success rate across all observed prey captures [50].

### Predator models

We used life-sized models of four African carnivores (S1 Fig): lion (ears-feet 1.05 m x nose-tail 2.2 m), spotted hyena (0.9 m x 1.3 m), cheetah (0.9 m x 1.5 m), and wild dog (0.9 m x 1.3 m). Models of impala (horns-feet 1.2 m x nose-tail 0.9 m) and Thomson’s gazelle (1.1 m x 0.9 m) were employed as controls. High-quality photographs depicting each animal laterally were printed on a plywood base and cut to shape. We constructed an all-terrain trolley on which we could mount the models and move them through the bush parallel to nearby prey animals. Two different models of each species were employed.

#### Permit information

All experiments were conducted in accordance with the University of Minnesota Institutional Animal Care and Use Committee (IACUC protocol no. 1510- 33082A).

### Experimental protocol

Focal prey species were located by driving daily transects within each reserve. As these animals were not individually identifiable in the field, experiments performed on the same species were conducted at least 0.5 km apart on the same day and at least 24 h apart at the same location to minimize the possibilities of resampling and habituation. Sites were never sampled for more than three consecutive weeks but were occasionally resampled months or years apart (S1 Table). All study sites were popular tourist destinations receiving thousands of annual visitors and animals were strongly acclimated to human activities. We saw no evidence that our vehicle or presence affected the natural behavior of these animals during the trials.

After locating a focal group, a model was placed on the trolley and set behind natural cover within 100–200 m of the herd. We then drove 100–200 m while unspooling a rope attached to the trolley. After all animals had resumed their prior activities, the model was drawn towards the vehicle in full view of the group. The model therefore emerged from cover and traveled at a moderate pace (3–5 km/hour) parallel to the focal individuals. Each trial was filmed using a Lumix DMC-FZ70 camera (Panasonic; Osaka, Japan), beginning from the moment of model presentation until the herd had resumed its previous activity for 60s or moved out of sight. Local ecologists accompanied the research team for multiple trials to confirm whether the models evoked prey responses that were typical of real encounters between each predator-prey dyad.

We recorded the following variables during each trial: initial distance between the focal group and the model, focal group size and composition (age [adult or juvenile], sex [male, female, or unidentified], and the presence of heterospecific prey species), and the dominant habitat within a 50 m radius of the experiment (‘open’: grasses < 50 cm tall, minimal woody vegetation; ‘closed’: dense woody vegetation).

### Behavioral observations

Video recordings were scored blind to minimize bias and by a single observer to maximize consistency in response characterization. Three focal individuals were chosen from each trial, selected haphazardly from the left and right peripheries and the center of the group. At least one member of each sex was selected if the herd contained distinguishable males and females; juveniles were never used. For these individuals, we recorded i) presence of alarm-calling, flight, clumping, stotting (exaggerated leaps during flight), and tail flagging (prominent visual display of white tail), ii) duration of vigilance directed towards the model or surrounding environment, iii) frequency of alarm-calling, and iv) latency from the individual’s first detection of the model until its onset of flight or alarm-calling. Any case of snorting, whistling, barking, or braying elicited by the model was characterized as ‘alarm-calling’. ‘Clumping’ was defined as the act of a dispersed foraging group moving together to form a tight herd (< 1 m between individuals). Tail flagging only occurred in two instances, so this response was excluded from our analyses.

### Statistical analyses

Analyses were performed in Program R, v. 3.5.3 [52]. Continuous variables were centered and scaled relative to their means and standard deviations, respectively. We fit the following statistical models with interactions between prey species and each predator parameter: hunting style, capture success, prey preference, and relative predator density. For the control trials, predator risk values (capture success, density) were set to zero and prey preference was set to -1 (e.g., would never pose a threat to the prey species). For all tests, we included model height (i.e., detectability from a distance), habitat type, initial distance between model and focal individual, focal herd size, and the presence of juveniles and heterospecific prey species as additional explanatory variables and performed backwards stepwise selection on these non-interacting covariates to improve statistical fit and reduce residual spread. Unless noted otherwise, we used focal individual data as our response for all tests and incorporated random effects of experimental trial and study reserve to account for nested data. For generalized linear mixed models (GLMMs), we performed post-hoc comparisons on interacting factors using FDR-adjusted p values for multiple tests (“contrasts”; package ‘emmeans’ [53]). Results were considered strongly supported where 95% confidence intervals did not overlap 0 (simple slopes) or adjusted-α ≤ 0.05 (simple effects). Results are given on log-odds ratio scale unless otherwise noted.

#### Type of response

We analyzed the presence of flight, alarm-calling, clumping (wildebeest and zebra), and stotting (impala) during experimental trials using GLMMs fit with logit-link functions and binomial error distributions (function ‘glmmTMB’, package ‘glmmTMB’ [54]). Clumping behavior was evaluated at the whole-group level, thus the random effect for focal individual was not used, and we only included trials containing more than one individual (retaining 68.0% of wildebeest trials and 96.9% of zebra trials) to allow for the possibility of clumping. Statistical model fit was evaluated by examination of simulated scaled residuals and dispersion parameters (package ‘DHARMa’ [55]).

#### Intensity of response

We employed two approaches to quantify response intensity. We fit GLMMs to duration of vigilance (zero-inflated negative binomial distribution with log-link function) and number of alarm-calls (zero-inflated Poisson distribution with log-link function). These tests included an offset for the total time of the encounter and were assessed as described above. Latency to flee and alarm-call were analyzed using mixed-effects Cox proportional hazard models (function ‘coxme’, package ‘coxme’ [56]), which assess the effects of multiple variables on the onset of each event. If the behavior did not occur, the response time was set equivalent to the length of the observation and the event was right censored. Hazard ratios (HRs) for each covariate indicate whether the variable is positively (HR > 1) or negatively (HR < 1) associated with event probability (e.g., either sooner or later, respectively). Model fit was evaluated using the global model Wald and chi-square statistics.

## Results

### Sample size

We conducted a total of 365 experiments, exposing an average 24.3 herds of each prey species to each type of predator or control model (herds per model: mean_impala_ = 29.6, range_impala_ = 17–44; mean_wildebeest_ = 23.8, range_wildebeest_ = 8–31; mean_zebra_ = 19.6, range_zebra_ = 8–27; S2 Table). Experiments were conducted across a range of social and environmental contexts (S3 Table).

### Drivers of anti-predator behavior

**Fig 2:**
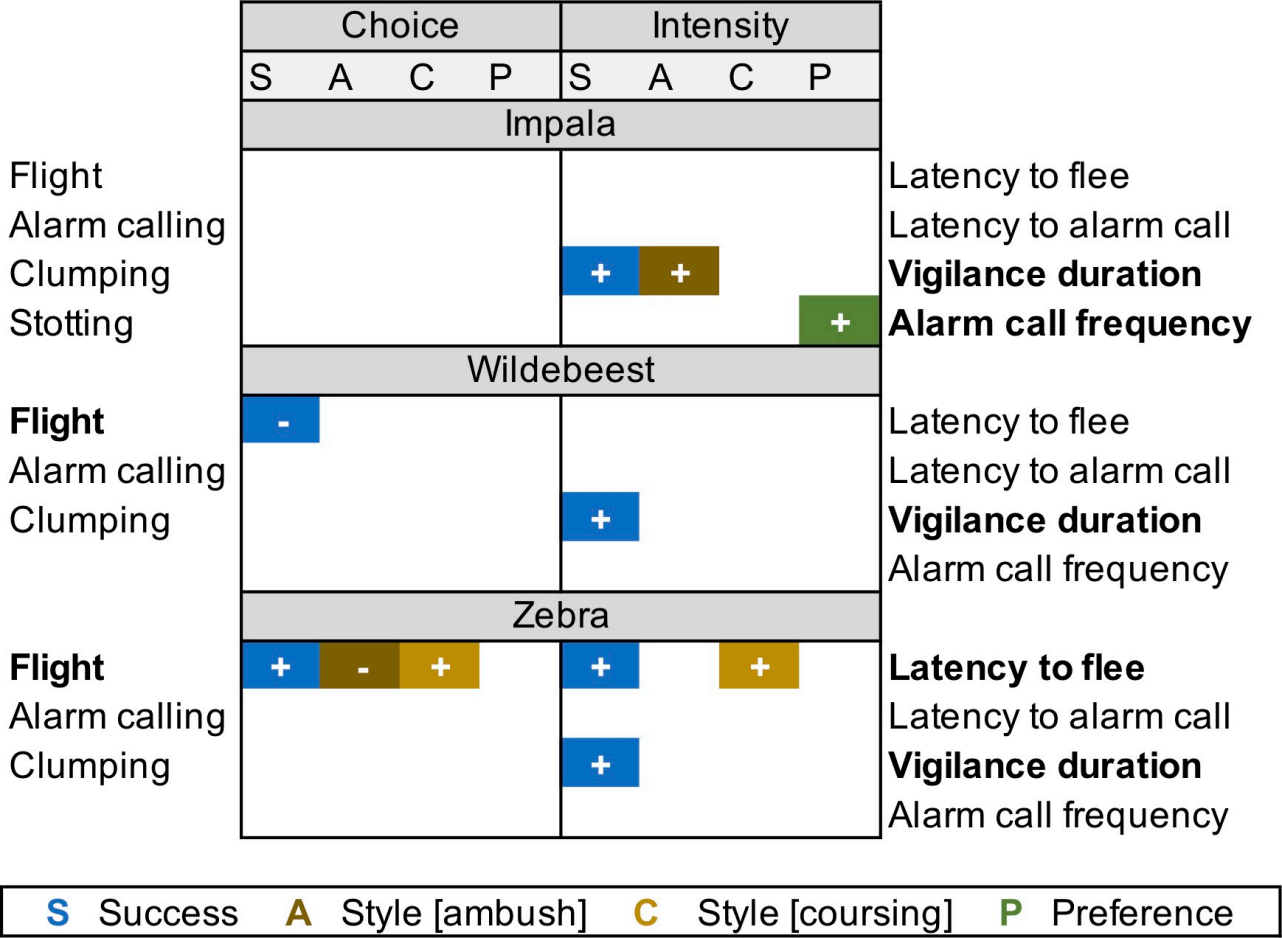
Anti-predator behavioral strategies adopted by focal prey species relative to predator traits. **Impala** relied on the same suite of behaviors, modulating the intensity of their response relative to multiple predator traits. **Wildebeest** altered the type and intensity of response, but only responded to predator capture success. **Zebra** adjusted both the type and intensity of behaviors performed in response to multiple characterizations of threat. Colored blocks correspond to predator traits that were strongly supported to affect response performance with +/- indicating the direction of the response. Relative predator density was never a significant driver of anti-predator tactics and is therefore unlisted.

**Fig 3:**
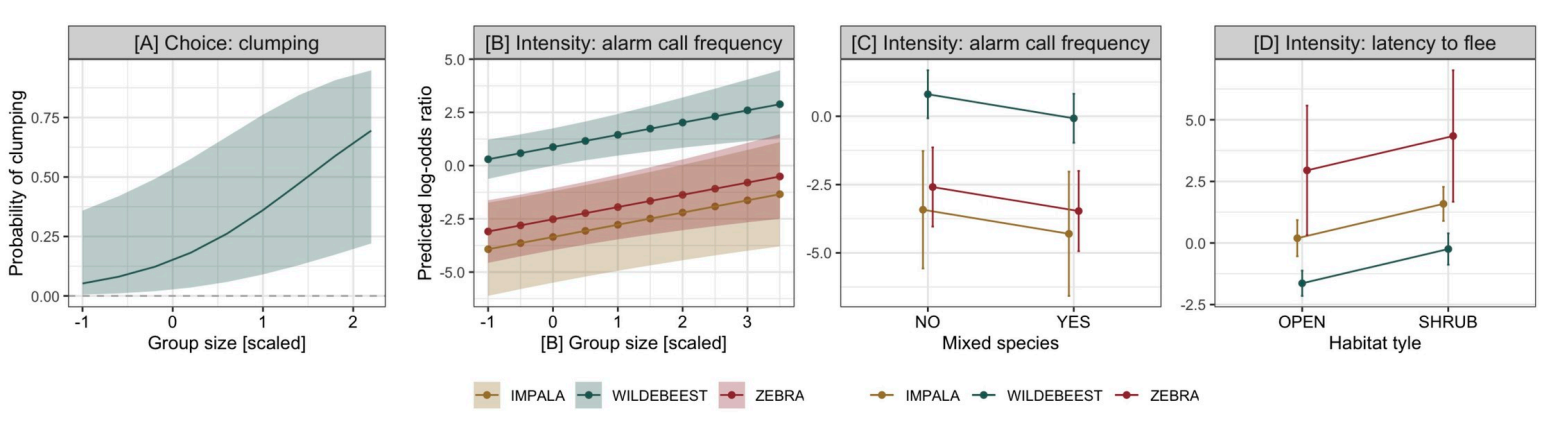
Strongly supported effects of social and environmental variables on anti-predator decision-making. **[A]** Wildebeest were more likely to form defensive clumps when more conspecifics were present. In all three focal species **[B]** alarm-calling frequency increased with group size, **[C]** alarm-calling frequency decreased when heterospecifics were present, and **[D]** flight occurred sooner in experimental trials that took place in closed habitats. Baseline performance of these behaviors was higher in wildebeest than in zebra and impala for alarm-call frequency **[B, C]** and lower for latency to flee **[D]**. Probability of behavioral response and log-odds ratio for choice **[A]** and intensity **[B-D]** of responses, respectively, are depicted with associated 95% CIs.

Across prey species, all predator characteristics except density influenced reactive anti-predator strategies (Fig 2). In terms of *type* of response, zebra and wildebeest altered their propensity to flee based on the hunting style and capture success of each predator. Anti-predator behaviors used by impala, however, were similar across predators. For response *intensity*, prey modulated their behavioral performance based on predator capture success and either hunting style (impala, zebra) or prey preference (wildebeest). Capture success was therefore the only risk characterization that broadly predicted anti-predator responses across prey species. Prey species-specific responses involved modulating vigilance, flight, and/or alarm-calling in response to specific aspects of predation threat (S4–S9 Tables), with some prey performing certain anti-predator behaviors more than others. Herd size, presence of heterospecifics, and habitat in which the encounter took place further shaped these tactics (Fig 3).

#### Type of response

**Fig 4:**
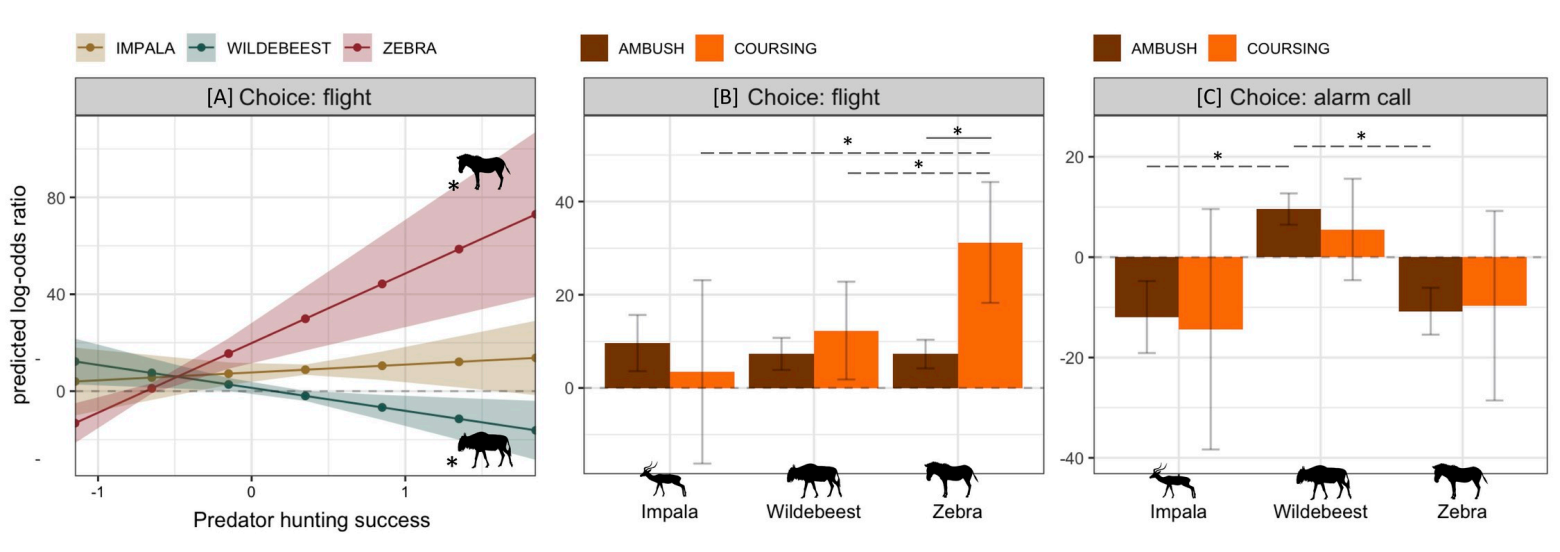
Strongly-supported effects on type of anti-predator behaviors chosen. **[A]** Zebra and wildebeest were more and less likely to flee, respectively, from increasingly successful predators. **[B]** Zebra were more likely to flee from coursing predators than ambush predators and fled more often from coursing predators than either impala or wildebeest did. **[C]** Wildebeest were increasingly likely to alarm-call in encounters with ambush predators than either impala or zebra. Depicted are log-odds ratios with associated 95% CIs. Ratios above and below 0 (dashed line) indicate that the outcome is more or less likely. For [A] and [C], solid significance lines indicate differences in response to stimuli within a species while dashed significance lines represent inter-species differences in response to the same stimulus.

Choice of anti-predator behavior in impala was not influenced by predator traits (all 95% CI contained 0 or adjusted-p > 0.05). For wildebeest and zebra, predator traits only affected the decision to flee (Fig 2; S4–S6 Tables). During encounters with the most successful hunters, flight probability increased for zebra (simple β = 28.74, SE = 7.13, 95% CI = [14.75, 42.75]) but decreased for wildebeest (simple β = -9.48, SE = 3.64, 95% CI = [-16.62, -2.34]) (Fig 4A). Zebra fled more often from coursing predators than ambush predators (contrast = 23.958, SE = 6.860, t ratio = 3.49, p < 0.001) and were overall more likely to flee when confronting coursing predators than either wildebeest (contrast = 18.921, SE = 7.750, t ratio = 2.44, p = 0.015) or impala (contrast = 27.788, SE = 11.840, t ratio = 2.35, p = 0.019) (Fig 4B). Wildebeest, in contrast, were more likely to alarm-call towards ambush predators than the other prey species (impala: contrast = 21.520, SE = 3.920, t ratio = 5.49, p = < 0.001; zebra: contrast = 20.349, SE = 2.860, t ratio = 7.12, SE = < 0.001) but not more than they called towards coursing predators (Fig 4C).

Social context affected herding propensity for wildebeest and zebra, where larger herds clumped more often (β = 1.190, SE = 0.414, z value = 0.29, p = 0.004) (Fig 3A). Impala never exhibited clumping behavior.

#### Intensity of response

**Fig 5:**
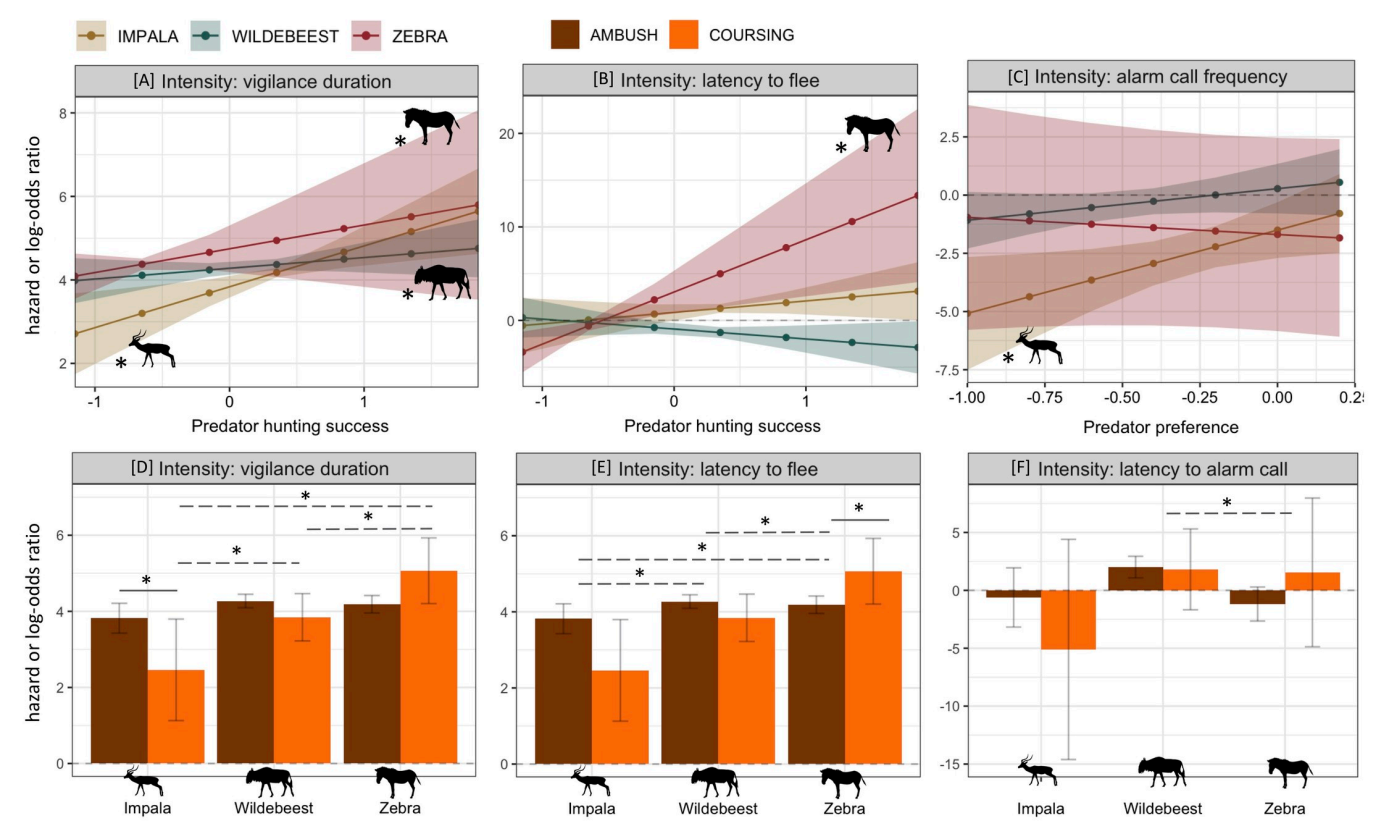
Strongly supported predator trait effects on intensity of anti-predator response. **[A]** All species were increasingly vigilant when encountering successful hunters. **[B]** Zebra fled sooner from encounters with successful predators. **[C]** Impala alarm-called more frequently towards predators that preferentially hunted impala. **[D]** Impala directed longer periods vigilance towards ambush predators than coursing predators, while zebra were more vigilant facing coursing predators than either of the other two species. **[E]** Zebra fled sooner from coursing predators than ambush predators and fled encounters with coursing predators earlier than wildebeest or impala. Impala fled sooner from ambush predators than other species did. **[F]** When encountering ambush predators, wildebeest alarm-called sooner than zebra. Depicted are log-odds (duration, vigilance) or hazard (latencies) ratios with associated 95% CIs. Ratios above and below 0 (dashed line) indicate that the outcome is more or less likely to occur, respectively. Solid significance lines represent differences in response to stimuli within a single species while dashed significance lines represent inter-species differences in response to the same stimulus.

Vigilance duration, frequency of alarm-calls, and latency to flee were affected by predator capture success, prey preference, and hunting style (Fig 2; S7–S9 Tables). Models of predators with higher capture success were monitored for longer periods by all focal species (β = 0.979, SE = 0.336, z value = 2.91, p = 0.004) (Fig 5A). This relationship was strongest for impala (simple β = 0.979 compared to simple β_wildebeest_ = 0.257 and simple β_zebra_ = 0.569). When flight occurred, zebra fled sooner from more successful predators (simple β = 5.580, SE = 1.936, 95% CI = [1.786, 9.377]) (Fig 5B). When encountering predators that had strong selective preferences for the focal prey, impala increased their alarm-call frequency (simple β = 3.575, SE = 1.58, 95% CI = [0.473, 6.680]) (Fig 5C). When comparing responses to ambush vs. coursing predators, impala monitored ambush predators longer (simple β = 1.357, SE = 0.552, z value = 2.460, p = 0.014) (Fig 5D) whereas zebra fled sooner from coursing predators (simple β = 5.241, SE = 1.925, z value = 2.722, p = 0.007) (Fig 5E). Impala fled sooner from ambush predators than either wildebeest (contrast = 2.284, SE = 0.673, t ratio = 3.40, p = 0.001) or zebra (contrast = 1.877, SE = 0.724, t ratio = 2.59, p = 0.010) (Fig 5E). Wildebeest alarm-called sooner when confronted with ambush predators than did zebra (contrast = 3.192, SE = 0.908, t ratio = 3.514, p = < 0.001) (Fig 5F) and were more vigilant towards ambush predators than impala (contrast = 0.452, SE = 0.219, t ratio = 2.066, p = 0.039) (Fig 5D). Zebra, on the other hand, fled sooner from coursing predators than did wildebeest (contrast = 4.723, SE = 2.162, t ratio = 2.19, p = 0.029) or impala (contrast = 5.470, SE = 2.699, t ratio = 2.03, p = 0.043) (Fig 5E) and remained vigilant for longer periods with coursing predators than did the other prey species (wildebeest: contrast = 1.223, SE = 0.542, t ratio = 2.26, p = 0.024; impala: contrast = 2.606, SE = 0.810, t ratio = 3.22, p = 0.036) (Fig 5D).

Social and environmental context modulated the intensity of anti-predator responses across all species (S6 Table). Alarm-calling frequency increased with herd size (β = 0.521, SE = 0.176, z value = 2.97, p = 0.003; Fig 3B) but decreased in the presence of heterospecifics (β = -1.000, SE = 0.339, z value = -2.95, p = 0.003; Fig 3C). Prey were swifter to flee (hazard ratio = 3.594, SE = 0.245, z value = 5.22, p < 0.001) if the encounter occurred in closed habitats (Fig 3D).

## Discussion

Systematic evaluation of reactive anti-predator responses to predator functional diversity allows us to refine our frameworks for understanding how predators shape prey behavior. We had predicted that choice of anti-predator response would be driven by predator hunting style whereas the perceived level of predation risk would influence the intensity at which the response was performed. Instead, we found that predator hunting style, success rate, and prey preference all played a role in determining overall risk-mitigation strategies of impala, wildebeest and zebra. Capture success was the only trait that elicited a universal response (increased vigilance) across species. Otherwise, prey reacted to aspects of predation threat in distinct ways. Impala performed the same suite of behaviors in response to all four predators but modulated the intensity of their responses according to particular predator traits. Both wildebeest and zebra altered the type and intensity of their response, but while wildebeest behavior was driven only by capture success, zebra tactics were shaped by multiple predator traits. Response intensity was further modified by social and environmental context, highlighting how multiple factors interact with predator traits to shape overall anti-predator strategies [57–59].

Prey altered aspects of flight, alarm-calling, and vigilance behavior in response to predator hunting style. Alerting and vigilance behaviors were primarily directed towards ambush predators and evasion used in response to coursing predators, lending support to hypothesized functions of these behaviors (i.e., signaling the detection of ambush predators to remove the element of surprise vs. gaining a ‘head start’ on coursing predators [20, 21]; Table 1). Impala and wildebeest modulated the intensity of vigilance (both) and warning behaviors (wildebeest) when faced with ambush predators. Zebra fled more often and earlier from coursing predators. Among prey species, we also see differences in the baseline propensity to react to predators of different hunting styles. Latency to flee and alarm call was lower in impala and wildebeest, respectively, when faced with ambush predators than either of the other species. Wildebeest were more vigilant in general towards ambush predators while zebra fled sooner and displayed longer periods of vigilance in encounters with coursing predators compared to other prey. These contrasts may reflect the natural history of each species. Coursing predators typically select the vulnerable and sick, while ambush predators more often capture healthier individuals [21, 43]. For zebra, which live in closely-related family units [60], the optimal response may be to immediately evacuate the entire kin group from the area. Wildebeest and impala typically live in larger herds that contain fewer close relatives [11], and, thus, minimizing the individual risk of being selected by an ambush predator may take precedence. In this case, we might predict a reduction in flight responses that make an individual stand out or inadvertently move within reach of a hidden lion and more actions that maintain a ‘safety in numbers,’ such as vigilance and monitoring [61, 62].

The relative level of perceived risk was further expected to shape anti-predator behavioral responses. We deconstructed “risk” into proxies for each stage of the predation process: relative predator density, the preference of each predator for each prey species, and prey-species-specific capture success [13, 29]. That density never influenced anti-predator strategy suggests that high contact rates between predators and prey alone is not sufficient for prey to evaluate predators as “risky” [29] or that the distribution of predators or of areas where predators experience high hunting success may be more important than overall predator abundance [63, 64]. Predator selectivity was only important to impala, which increased their alarm-calling rate in response to species that prefer to hunt them. Smaller prey species are at risk from larger suites of predators [65, 66]. Impala may therefore face novel trade-offs between i) performing specific responses that alleviate risk from one type of predator while potentially increasing risk from another [18, 67] and ii) more generalized responses which may simultaneously reduce risk from a broad range of threats [15].

Prey universally responded to the most successful predators by increasing their levels of vigilance. Zebra were also more likely to flee and fled sooner during encounters with highly lethal predators. Wildebeest, were less likely to flee from these threats, instead appearing to offset this response by increasing the amount of time spent monitoring the danger. The use of complementary anti-predator responses such as this would not have been detected if we had only examined a single behavioral metric (see [68–70]). Several authors have noted that anti-predatory behaviors should decrease levels of direct predation to the point that lethality becomes a poor proxy for predation threat [64, 71], and studies in other large herbivore communities found that capture success fails to predict proactive anti-predator avoidance behaviors (e.g., [72]) and reactive levels of vigilance to predators within 0.5 km [59, 69]. However, our methodology measured responses to risk at a different, scale-dependent stage within the predation process. Strategies that decouple lethality from behavioral performance at broader spatiotemporal scales (e.g., predation events per predator per day) would be expected to differ from those that are a reaction to the imminent risk of being killed during a particular hunt (e.g., predation events per predator per encounter).

The physical and social environments may also shape anti-predator decision-making [57, 68, 70]. The distribution of resources across the landscape differs for browsers and grazers, forcing certain species to enter habitats of varying complexity [59] and/or that differ in underlying predation risk [57]. All prey fled and alarm-called more frequently in dense habitats, suggesting that large herbivores perceive these habitats as particularly dangerous [73–75]. Social environment was equally important. Advantages of clumping and communicating predator presence appeared to increase with the number of nearby conspecifics, and alarm-call rates dropped when heterospecific prey were in the immediate vicinity. Decreased risk perception driven by the ‘dilution’ [62] or ‘many eyes’ [61] effects or eavesdropping [76] may be particularly strong when prey are surrounded by species that are more sensitive to or preferred by predators [23, 77, 78].

Direct encounters between predators and prey are only rarely observed in the wild, and much research on reactive anti-predator responses has assumed that predators and prey located within a certain radius have knowledge of each other’s presence (e.g., [59, 68, 70, 79]). By simulating face-to-face encounters with ‘predators’, we were able to generate a sufficient number of predator-prey interactions to draw inference on mechanisms underlying anti-predator strategies. Model predators have previously been used to elicit anti-predator behavior in a variety of animals (e.g., fish [80]; lizards [81], rodents [82–83], primates [84]) including large herbivores [85]. We used visual rather than auditory cues (e.g., playbacks) as most carnivores do not vocalize while hunting [84] and herbivores in our system primarily rely on vision to detect predators [22]. However, we acknowledge that predator detection is a multi-modal process and that our models lacked behavioral signals of intent such as mobile gaze and posture that would allow prey to update their assessments of risk ([14, 43, 86] but see [17]). Given their constant horizontal motion and unchanging postures, our models might not have triggered maximal fear responses if the prey did not perceive them as actively hunting, particularly the ambush predators.

By complementing recent work characterizing how prey may proactively avoid encountering predators, we have related reactive risk mitigation tactics, within and across prey species, to elements of predator functional diversity [18, 20, 59, 70]. Prey behavior is shaped by interactions between chronic and imminent predation threat (e.g., [79]); identifying responses to acute risk is key for building an integrative framework that allows us to predict predator effects on prey behavior across spatiotemporal scales.

## Supporting information

S1 Fig One exemplar of each life-sized predator model. Photorealistic predator models were mounted on all-terrain trolleys which were used to move predators through the bush towards prey individuals, simulating a predator encounter. Control models of impala and Thomson’s gazelle not pictured.
(DOCX)

S1 Table Sampling dates for each. Sites were never sampled for more than three consecutive weeks in order to avoid habituating focal animals (note: the third sampling period for the Phinda/Thanda complex was greater than three weeks but split between two separate reserves).
(DOCX)

S2 Table Distribution of experimental trials by prey species, predator model, and study site. Note that prey individuals were only tested with models of predators present in their reserve (i.e., that they had ecological experience with).
(DOCX)

S3 Table Distribution of experimental conditions. Summary of social and environmental context under which experiments took place for each species. Reported are focal individual counts (group-level counts). Not all of the measured variables made it into top-ranking models. Range of model presentation distances is 2–142 m (mean = 70.1 m, median = 73 m). Range of impala herd sizes is 1–43 individuals (mean = 12.0 individuals, median = 9 individuals). Range of wildebeest herd sizes is 1–32 (mean = 8.8, median = 7). Range of zebra herd sizes is 1–19 (mean = 6.5, median = 5).
(DOCX)

S4 Table Choice of response. GLMM results for presence of prey anti-predator response (flight, alarm calling, grouping, and stotting) during encounters with predator models. Note that the interaction effect tests the difference between simple slopes (continuous) or effects (categorial), not whether each simple slope/effect is different from 0. Post hoc tests (see S4 and S5 Tables) are used to evaluate differences between and support for interacting variables. For these models, the reference level for prey species in impala, for habitat is open habitat, and for hunting strategy is the control model.
(DOCX)

S5 Table Choice of response. Post-hoc interaction analysis of GLMM results from S3 Table using package ‘emmeans’ [83]. (A) Simple slopes (estimates of slopes of the covariate trend of each level of the factor; continuous covariates) and (B) simple effects (general contrasts of factor levels; categorical covariates) are presented for each prey species.
(DOCX)

S6 Table Choice of response. Post-hoc interaction analysis of GLMM results from S3 Table using package ‘emmeans’ [83], evaluating the pairwise differences between species responses (i.e., conditional contrasts) for (A) continuous and (B) categorical predictors.
(DOCX)

S7 Table Intensity of response. GLMM results for intensity of prey anti-predator response (vigilance duration, alarm call frequency, latency to flee, and latency to alarm call) during encounters with predator models. Note that the interaction effect tests the difference between simple slopes (continuous) or effects (categorial), not whether each simple slope/effect is different from 0. Post hoc tests (see S7 and S8 Tables) are used to evaluate differences between and support for interacting variables. For these models, the reference level for prey species in impala, for habitat is open habitat, and for hunting strategy is the control model.
(DOCX)

S8 Table Intensity of response. Post-hoc interaction analysis of GLMM results from S6 Table using package ‘emmeans’ [83]. (A) Simple slopes (estimates of slopes of the covariate trend of each level of the factor; continuous covariates) and (B) simple effects (general contrasts of factor levels; categorical covariates) are presented for each prey species.
(DOCX)

S9 Table Intensity of response. Post-hoc interaction analysis of GLMM results from S6 Table using package ‘emmeans’ [83], evaluating the pairwise differences between species responses (i.e., conditional contrasts) for (A) continuous and (B) categorical predictors.
(DOCX)
