# Supplementary material for: Reactive anti-predator behavioral strategy shaped by predator characteristics
Source: PLoS One. 2021 Aug 18;16(8):e0256147. doi: 10.1371/journal.pone.0256147 (PMC8372962; doi:10.1371/journal.pone.0256147)
Supplement: S1 Fig — Photorealistic predator models were mounted on all-terrain trolleys which were used to move predators through the bush towards prey individuals, simulating a predator encounter. Control models of impala and Thomson’s gazelle not pictured. (DOCX) [file pone.0256147.s001.docx]

**“Reactive anti-predator behavioral strategy shaped by predator characteristics”**

**S1 Fig.** One exemplar of each life-sized predator model. Photorealistic predator models were mounted on all-terrain trolleys which were used to move predators through the bush towards prey individuals, simulating a predator encounter. Control models of impala and Thomson’s gazelle not pictured.
