# Supplementary material for: Reactive anti-predator behavioral strategy shaped by predator characteristics
Source: PLoS One. 2021 Aug 18;16(8):e0256147. doi: 10.1371/journal.pone.0256147 (PMC8372962; doi:10.1371/journal.pone.0256147)
Supplement: S1 Table — Sites were never sampled for more than three consecutive weeks in order to avoid habituating focal animals (note: the third sampling period for the Phinda/Thanda complex was greater than three weeks but split between two separate reserves). (DOCX) [file pone.0256147.s002.docx]

**“Reactive anti-predator behavioral strategy shaped by predator characteristics”**

**S1 Table.** Sampling dates for each. Sites were never sampled for more than three consecutive weeks in order to avoid habituating focal animals (note: the third sampling period for the Phinda/Thanda complex was greater than three weeks but split between two separate reserves).

| Reserve | Begin | End |
| --- | --- | --- |
| Phinda/Thanda | 7/27/15 | 8/3/15 |
| Phinda/Thanda | 10/15/16 | 10/23/16 |
| Phinda/Thanda | 6/30/16 | 7/30/16 |
| Pilanesberg | 6/9/16 | 6/24/16 |
| Pilanesberg | 8/3/16 | 8/8/16 |
| Tswalu | 6/12/15 | 6/27/15 |
