# Supplementary material for: Reactive anti-predator behavioral strategy shaped by predator characteristics
Source: PLoS One. 2021 Aug 18;16(8):e0256147. doi: 10.1371/journal.pone.0256147 (PMC8372962; doi:10.1371/journal.pone.0256147)
Supplement: S2 Table — Note that prey individuals were only tested with models of predators present in their reserve (i.e., that they had ecological experience with). (DOCX) [file pone.0256147.s003.docx]

**“Reactive anti-predator behavioral strategy shaped by predator characteristics”**

**S2 Table.** Distribution of experimental trials by prey species, predator model, and study site. Note that prey individuals were only tested with models of predators present in their reserve (i.e., that they had ecological experience with).

| Prey species | Predator Model | Reserve | Total Encounters |
| --- | --- | --- | --- |
| Impala | Control | Phinda/Thanda | 27 |
| Impala | Control | Pilanesberg | 17 |
| Impala | Hyena | Phinda/Thanda | 22 |
| Impala | Wild dog | Pilanesberg | 16 |
| Impala | Wild dog | Tswalu | 1 |
| Impala | Cheetah | Phinda/Thanda | 20 |
| Impala | Cheetah | Pilanesberg | 14 |
| Impala | Lion | Phinda/Thanda | 21 |
| Impala | Lion | Pilanesberg | 10 |
| Wildebeest | Control | Phinda/Thanda | 10 |
| Wildebeest | Control | Pilanesberg | 14 |
| Wildebeest | Control | Tswalu | 5 |
| Wildebeest | Hyena | Phinda/Thanda | 8 |
| Wildebeest | Wild dog | Pilanesberg | 20 |
| Wildebeest | Wild dog | Tswalu | 6 |
| Wildebeest | Cheetah | Phinda/Thanda | 9 |
| Wildebeest | Cheetah | Pilanesberg | 16 |
| Wildebeest | Cheetah | Tswalu | 6 |
| Wildebeest | Lion | Phinda/Thanda | 14 |
| Wildebeest | Lion | Pilanesberg | 11 |
| Zebra | Control | Phinda/Thanda | 15 |
| Zebra | Control | Pilanesberg | 11 |
| Zebra | Hyena | Phinda/Thanda | 17 |
| Zebra | Wild dog | Pilanesberg | 8 |
| Zebra | Cheetah | Phinda/Thanda | 11 |
| Zebra | Cheetah | Pilanesberg | 9 |
| Zebra | Lion | Phinda/Thanda | 19 |
| Zebra | Lion | Pilanesberg | 8 |
