# Supplementary material for: Reactive anti-predator behavioral strategy shaped by predator characteristics
Source: PLoS One. 2021 Aug 18;16(8):e0256147. doi: 10.1371/journal.pone.0256147 (PMC8372962; doi:10.1371/journal.pone.0256147)
Supplement: S3 Table — Summary of social and environmental context under which experiments took place for each species. Reported are focal individual counts (group-level counts). Not all of the measured variables made it into top-ranking models. Range of model presentation distances is 2–142 m (mean = 70.1 m, median = 73 m). Range of impala herd sizes is 1–43 individuals (mean = 12.0 individuals, median = 9 individuals). Range of wildebeest herd sizes is 1–32 (mean = 8.8, median = 7). Range of zebra herd sizes is 1–19 (mean = 6.5, median = 5). (DOCX) [file pone.0256147.s004.docx]

**“Reactive anti-predator behavioral strategy shaped by predator characteristics”**

**S3 Table. Distribution of experimental conditions.** Summary of social and environmental context under which experiments took place for each species. Reported are focal individual counts (group-level counts). Not all of the measured variables made it into top-ranking models. Range of model presentation distances is 2-142 m (mean = 70.1 m, median = 73 m). Range of impala herd sizes is 1-43 individuals (mean = 12.0 individuals, median = 9 individuals). Range of wildebeest herd sizes is 1-32 (mean = 8.8, median = 7). Range of zebra herd sizes is 1-19 (mean = 6.5, median = 5).

|  | Impala | Wildebeest | Zebra |
| --- | --- | --- | --- |
| Habitat [open] | 124 (47) | 211 (93) | 175 (64) |
| Habitat [closed] | 253 (101) | 51 (26) | 85 (34) |
| Mixed species [present] | 155 (57) | 110 (49) | 148 (55) |
| Mixed species [absent] | 222 (91) | 152 (70) | 112 (43) |
| Juveniles [present] | 375 (147) | 214 (103) | 190 (74) |
| Juveniles [absent] | 2 (1) | 48 (16) | 70 (24) |
| Model distance [< 70 m] | 186 (76) | 134 (57) | 114 (43) |
| Model distance [>= 70 m] | 191 (72) | 128 (62) | 146 (55) |
| Herd size [< avg. herd size] | (97) | (86) | (67) |
| Herd size [>= avg. herd size] | (51) | (33) | (31) |
