# Supplementary material for: Reactive anti-predator behavioral strategy shaped by predator characteristics
Source: PLoS One. 2021 Aug 18;16(8):e0256147. doi: 10.1371/journal.pone.0256147 (PMC8372962; doi:10.1371/journal.pone.0256147)
Supplement: S4 Table — GLMM results for presence of prey anti-predator response (flight, alarm calling, grouping, and stotting) during encounters with predator models. Note that the interaction effect tests the difference between simple slopes (continuous) or effects (categorial), not whether each simple slope/effect is different from 0. Post hoc tests (see S4 and S5 Tables) are used to evaluate differences between and support for interacting variables. For these models, the reference level for prey species in impala, for habitat is open habitat, and for hunting strategy is the control model. (DOCX) [file pone.0256147.s005.docx]

**“Reactive anti-predator behavioral strategy shaped by predator characteristics”**

**S4 Table. Choice of response.** GLMM results for presence of prey anti-predator response (flight, alarm calling, grouping, and stotting) during encounters with predator models. Note that the interaction effect tests the difference between simple slopes (continuous) or effects (categorial), not whether each simple slope/effect is different from 0. Post hoc tests (see Table S4, S5) are used to evaluate differences between and support for interacting variables. For these models, the reference level for prey species in impala, for habitat is open habitat, and for hunting strategy is the control model.

|  | Coefficient | Estimate | SE | z value | p value |
| --- | --- | --- | --- | --- | --- |
| Decision to flee | Strategy [ambush] | -1.572 | 12.672 | -0.12 | 0.901 |
|  | Strategy [coursing] | -7.760 | 19.889 | -0.39 | 0.696 |
|  | Prey [wildebeest] | -28.060 | 13.987 | -2.01 | 0.045 |
|  | Prey [zebra] | 17.545 | 13.834 | 1.27 | 0.205 |
|  | Density | -0.555 | 2.299 | -0.24 | 0.809 |
|  | Preference | 0.487 | 5.657 | 0.09 | 0.931 |
|  | Success | 3.249 | 4.940 | 0.66 | 0.511 |
|  | Habitat [closed] | 2.116 | 1.131 | 1.87 | 0.061 |
|  | Strategy [ambush] x Prey [wildebeest] | 27.768 | 14.592 | 1.90 | 0.057 |
|  | Strategy [coursing] x Prey [wildebeest] | 38.958 | 22.712 | 1.72 | 0.086 |
|  | Strategy [ambush] x Prey [zebra] | -22.163 | 15.646 | -1.42 | 0.157 |
|  | Strategy [coursing] x Prey [zebra] | 7.984 | 20.192 | 0.40 | 0.693 |
|  | Density x Prey [wildebeest] | 0.043 | 2.801 | 0.02 | 0.988 |
|  | Density x Prey [zebra] | 0.130 | 3.230 | 0.04 | 0.968 |
|  | Preference x Prey [wildebeest] | 1.238 | 6.886 | 0.18 | 0.857 |
|  | Preference x Prey [zebra] | 1.583 | 7.023 | 0.23 | 0.822 |
|  | Success x Prey [wildebeest] | -12.725 | 6.079 | -2.09 | 0.036 |
|  | Success x Prey [zebra] | 25.495 | 8.750 | 2.91 | 0.004 |
| Decision to alarm call | Strategy [ambush] | -4.169 | 14.505 | -0.29 | 0.774 |
|  | Strategy [coursing] | -6.593 | 23.295 | -0.28 | 0.777 |
|  | Prey [wildebeest] | 0.905 | 14.007 | 0.06 | 0.949 |
|  | Prey [zebra] | -3.127 | 17.387 | -0.18 | 0.857 |
|  | Density | 0.728 | 2.385 | 0.31 | 0.760 |
|  | Preference | 2.692 | 4.992 | 0.54 | 0.590 |
|  | Success | 0.821 | 6.650 | 0.12 | 0.902 |
|  | Juveniles [present] | -0.379 | 1.570 | -0.24 | 0.809 |
|  | Mixed species [present] | -0.961 | 1.183 | -0.81 | 0.417 |
|  | Strategy [ambush] x Prey [wildebeest] | 20.222 | 15.511 | 1.30 | 0.192 |
|  | Strategy [coursing] x Prey [wildebeest] | 18.586 | 25.227 | 0.74 | 0.461 |
|  | Strategy [ambush] x Prey [zebra] | 3.059 | 20.380 | 0.15 | 0.881 |
|  | Strategy [coursing] x Prey [zebra] | 6.573 | 23.993 | 0.27 | 0.784 |
|  | Density x Prey [wildebeest] | -1.398 | 2.894 | -0.48 | 0.629 |
|  | Density x Prey [zebra] | -0.263 | 3.818 | -0.07 | 0.945 |
|  | Preference x Prey [wildebeest] | -0.973 | 6.176 | -0.16 | 0.875 |
|  | Preference x Prey [zebra] | -2.702 | 6.423 | -0.42 | 0.674 |
|  | Success x Prey [wildebeest] | 1.229 | 7.408 | 0.17 | 0.868 |
|  | Success x Prey [zebra] | 0.453 | 12.148 | 0.04 | 0.970 |
| Decision to group [wildebeest, zebra] | Strategy [ambush] | -2.253 | 2.530 | -0.89 | 0.373 |
|  | Strategy [coursing] | -4.853 | 4.235 | -1.15 | 0.252 |
|  | Prey [zebra] | -1.803 | 78000.313 | 0.00 | 1.000 |
|  | Density | 0.836 | 0.767 | 1.09 | 0.276 |
|  | Preference | 0.364 | 1.321 | 0.28 | 0.783 |
|  | Success | 1.957 | 1.391 | 1.41 | 0.159 |
|  | Herd size | 1.190 | 0.414 | 2.88 | 0.004 |
|  | Mixed species [present] | -0.999 | 0.569 | -1.76 | 0.079 |
|  | Strategy [ambush] x Prey [zebra] | 2.655 | 87204.466 | 0.00 | 1.000 |
|  | Strategy [coursing] x Prey [zebra] | -14.043 | 31110.591 | 0.00 | 1.000 |
|  | Density x Prey [zebra] | -1.067 | 1.226 | -0.87 | 0.384 |
|  | Preference x Prey [zebra] | 0.400 | 3427.079 | 0.00 | 1.000 |
|  | Success x Prey [zebra] | -1.223 | 65006.206 | 0.00 | 1.000 |
| Stot [impala] | Strategy [ambush] | 2.881 | 15.48 | 0.19 | 0.852 |
|  | Strategy [coursing] | 4.505 | 24.349 | 0.19 | 0.853 |
|  | Density | -0.112 | 2.934 | -0.04 | 0.969 |
|  | Preference | -0.592 | 6.115 | -0.10 | 0.923 |
|  | Success | -1.265 | 6.427 | -0.20 | 0.844 |
