# Supplementary material for: Reactive anti-predator behavioral strategy shaped by predator characteristics
Source: PLoS One. 2021 Aug 18;16(8):e0256147. doi: 10.1371/journal.pone.0256147 (PMC8372962; doi:10.1371/journal.pone.0256147)
Supplement: S5 Table — Post-hoc interaction analysis of GLMM results from S3 Table using package ‘emmeans’ [83]. (A) Simple slopes (estimates of slopes of the covariate trend of each level of the factor; continuous covariates) and (B) simple effects (general contrasts of factor levels; categorical covariates) are presented for each prey species. (DOCX) [file pone.0256147.s006.docx]

**“Reactive anti-predator behavioral strategy shaped by predator characteristics”**

**S5 Table. Choice of response.** Post-hoc interaction analysis of GLMM results from Table S3 using package ‘emmeans’ [83]. (A) Simple slopes (estimates of slopes of the covariate trend of each level of the factor; continuous covariates) and (B) simple effects (general contrasts of factor levels; categorical covariates) are presented for each prey species.

(A) Simple slopes

|  | Predictor | Species | Simple slopes | SE | 95% CI |
| --- | --- | --- | --- | --- | --- |
| Probability of flight | Density | Impala | -0.555 | 2.299 | (-5.067, 3.958) |
|  |  | Wildebeest | -0.512 | 1.657 | (-3.764, 2.741) |
|  |  | Zebra | -0.425 | 2.329 | (-4.995, 4.145) |
|  | Preference | Impala | 0.487 | 5.657 | (-10.615, 11.589) |
|  |  | Wildebeest | 1.726 | 3.844 | (-5.818, 9.270) |
|  |  | Zebra | 2.071 | 4.082 | (-5.94, 10.082) |
|  | Success | Impala | 3.249 | 4.940 | (-6.446, 12.944) |
|  |  | Wildebeest | -9.476 | 3.638 | (-16.616, -2.335) |
|  |  | Zebra | 28.743 | 7.129 | (14.752, 42.735) |
| Probability of alarm calling | Density | Impala | 0.724 | 2.371 | (-3.930, 5.377) |
|  |  | Wildebeest | -0.659 | 1.659 | (-3.916, 2.598) |
|  |  | Zebra | 0.439 | 3.020 | (-5.488, 6.367) |
|  | Preference | Impala | 2.722 | 4.974 | (-7.041, 12.485) |
|  |  | Wildebeest | 1.759 | 3.696 | (-5.495, 9.014) |
|  |  | Zebra | 0.062 | 4.031 | (-7.850, 7.974) |
|  | Success | Impala | 0.917 | 6.618 | (-12.072, 13.906) |
|  |  | Wildebeest | 2.079 | 3.382 | (-4.559, 8.716) |
|  |  | Zebra | 1.307 | 10.160 | (-18.633, 21.247) |
| prob. of clumping | Density | Impala | 0.836 | 0.767 | (-0.679, 2.351) |
|  |  | Wildebeest | -0.231 | 0.975 | (-2.155, 1.694) |
|  | Preference | Zebra | 0.364 | 1.321 | (-2.244, 2.972) |
|  |  | Impala | 0.765 | 3427.079 | (-6765.217, 6766.746) |
|  | Success | Wildebeest | 1.957 | 1.391 | (-0.788, 4.703) |
|  |  | Zebra | 0.734 | 65006.206 | (-128339.130, 128340.599) |

(B) Simple effects (hunting style)

|  | Levels | Species | Estimate | SE | t ratio | p value |
| --- | --- | --- | --- | --- | --- | --- |
| Probability of flight | Control | Impala | 3.429 | 7.519 | 0.456 | 0.648 |
|  |  | Wildebeest | -26.662 | 6.402 | -4.165 | 0.000 |
|  |  | Zebra | 23.233 | 7.239 | 3.210 | 0.002 |
|  | Ambush | Impala | 1.560 | 2.124 | 0.735 | 0.614 |
|  |  | Wildebeest | -0.762 | 1.509 | -0.505 | 0.614 |
|  |  | Zebra | -0.798 | 1.484 | -0.538 | 0.614 |
|  | Coursing | Impala | -12.218 | 7.241 | -1.687 | 0.138 |
|  |  | Wildebeest | -3.351 | 5.072 | -0.661 | 0.509 |
|  |  | Zebra | 15.570 | 5.522 | 2.819 | 0.015 |
| Probability of alarm calling | Control | Impala | -5.255 | 8.974 | -0.586 | 0.558 |
|  |  | Wildebeest | 12.435 | 6.900 | 1.802 | 0.216 |
|  |  | Zebra | -7.180 | 9.835 | -0.730 | 0.558 |
|  | Ambush | Impala | -7.587 | 2.526 | -3.004 | 0.003 |
|  |  | Wildebeest | 13.938 | 1.815 | 7.678 | 0.000 |
|  |  | Zebra | -6.351 | 2.008 | -3.163 | 0.002 |
|  | Coursing | Impala | -8.288 | 8.848 | -0.937 | 0.524 |
|  |  | Wildebeest | 11.640 | 6.208 | 1.875 | 0.183 |
|  |  | Zebra | -3.352 | 7.819 | -0.429 | 0.668 |
| Prob. of clumping | Control | Wildebeest | 1.067 | 36380.822 | 0.000 | 1.000 |
|  |  | Zebra | -1.067 | 36380.822 | 0.000 | 1.000 |
|  | Ambush | Wildebeest | -0.260 | 7221.411 | 0.000 | 1.000 |
|  |  | Zebra | 0.260 | 7221.411 | 0.000 | 1.000 |
|  | Coursing | Wildebeest | 8.089 | 22932.121 | 0.000 | 1.000 |
|  |  | Zebra | -8.089 | 22932.121 | 0.000 | 1.000 |
