# Supplementary material for: Reactive anti-predator behavioral strategy shaped by predator characteristics
Source: PLoS One. 2021 Aug 18;16(8):e0256147. doi: 10.1371/journal.pone.0256147 (PMC8372962; doi:10.1371/journal.pone.0256147)
Supplement: S6 Table — Post-hoc interaction analysis of GLMM results from S3 Table using package ‘emmeans’ [83], evaluating the pairwise differences between species responses (i.e., conditional contrasts) for (A) continuous and (B) categorical predictors. (DOCX) [file pone.0256147.s007.docx]

**“Reactive anti-predator behavioral strategy shaped by predator characteristics”**

**S6 Table. Choice of response.** Post-hoc interaction analysis of GLMM results from Table S3 using package ‘emmeans’ [83], evaluating the pairwise differences between species responses (i.e., conditional contrasts) for (A) continuous and (B) categorical predictors.

(A) Continuous predictors

|  | Predictor | Contrast | Estimate | SE | t ratio | p value |
| --- | --- | --- | --- | --- | --- | --- |
| Probability of flight | Density | Impala-Wildebeest | -0.043 | 2.801 | -0.015 | 1.000 |
|  |  | Impala-Zebra | -0.130 | 3.230 | -0.040 | 0.999 |
|  |  | Wildebeest-Zebra | -0.086 | 2.826 | -0.031 | 0.999 |
|  | Preference | Impala-Wildebeest | -1.238 | 6.886 | -0.180 | 0.982 |
|  |  | Impala-Zebra | -1.583 | 7.023 | -0.225 | 0.972 |
|  |  | Wildebeest-Zebra | -0.345 | 5.569 | -0.062 | 0.998 |
|  | Success | Impala-Wildebeest | 12.725 | 6.079 | 2.093 | 0.092 |
|  |  | Impala-Zebra | -25.495 | 8.750 | -2.914 | 0.010 |
|  |  | Wildebeest-Zebra | -38.219 | 8.599 | -4.445 | 0.000 |
| probability of alarm calling | Density | Impala-Wildebeest | 1.383 | 2.892 | 0.478 | 0.882 |
|  |  | Impala-Zebra | 0.284 | 3.825 | 0.074 | 0.997 |
|  |  | Wildebeest-Zebra | -1.098 | 3.444 | -0.319 | 0.946 |
|  | Preference | Impala-Wildebeest | 0.963 | 6.185 | 0.156 | 0.987 |
|  |  | Impala-Zebra | 2.660 | 6.388 | 0.416 | 0.909 |
|  |  | Wildebeest-Zebra | 1.697 | 5.453 | 0.311 | 0.948 |
|  | Success | Impala-Wildebeest | -1.162 | 7.397 | -0.157 | 0.986 |
|  |  | Impala-Zebra | -0.390 | 12.084 | -0.032 | 0.999 |
|  |  | Wildebeest-Zebra | 0.772 | 10.690 | 0.072 | 0.997 |
| p. clump. | Density | Wildebeest-Zebra | 1.067 | 1.226 | 0.870 | 0.385 |
|  | Preference | Wildebeest-Zebra | -0.400 | 3427.079 | 0.000 | 1.000 |
|  | Success | Wildebeest-Zebra | 1.223 | 65006.206 | 0.000 | 1.000 |

(B) Categorical predictors (hunting style)

|  | Contrast | Estimate | SE | t ratio | p value |
| --- | --- | --- | --- | --- | --- |
| Probability of flight | Impala [Control] - Impala [Ambush] | 1.545 | 12.355 | 0.125 | 0.901 |
|  | Impala [Control] - Impala [Coursing] | 7.890 | 19.383 | 0.407 | 0.684 |
|  | Impala [Control] - Wildebeest [Control] | 16.743 | 11.110 | 1.507 | 0.132 |
|  | Impala [Control] - Wildebeest [Ambush] | 4.466 | 9.820 | 0.455 | 0.649 |
|  | Impala [Control] - Wildebeest [Coursing] | 17.249 | 11.009 | 1.567 | 0.118 |
|  | Impala [Control] - Zebra [Control] | -18.304 | 12.883 | -1.421 | 0.156 |
|  | Impala [Control] - Zebra [Ambush] | 4.614 | 9.823 | 0.470 | 0.639 |
|  | Impala [Control] - Zebra [Coursing] | -18.552 | 11.472 | -1.617 | 0.106 |
|  | Impala [Ambush] - Impala [Coursing] | 6.345 | 7.969 | 0.796 | 0.426 |
|  | Impala [Ambush] - Wildebeest [Control] | 15.198 | 6.061 | 2.507 | 0.012 |
|  | Impala [Ambush] - Wildebeest [Ambush] | 2.921 | 3.485 | 0.838 | 0.402 |
|  | Impala [Ambush] - Wildebeest [Coursing] | 15.704 | 5.967 | 2.632 | 0.009 |
|  | Impala [Ambush] - Zebra [Control] | -19.849 | 9.122 | -2.176 | 0.030 |
|  | Impala [Ambush] - Zebra [Ambush] | 3.069 | 3.369 | 0.911 | 0.363 |
|  | Impala [Ambush] - Zebra [Coursing] | -20.097 | 6.968 | -2.884 | 0.004 |
|  | Impala [Coursing] - Wildebeest [Control] | 8.853 | 11.377 | 0.778 | 0.437 |
|  | Impala [Coursing] - Wildebeest [Ambush] | -3.424 | 10.064 | -0.340 | 0.734 |
|  | Impala [Coursing] - Wildebeest [Coursing] | 9.359 | 11.250 | 0.832 | 0.406 |
|  | Impala [Coursing] - Zebra [Control] | -26.194 | 13.039 | -2.009 | 0.045 |
|  | Impala [Coursing] - Zebra [Ambush] | -3.276 | 10.094 | -0.325 | 0.746 |
|  | Impala [Coursing] - Zebra [Coursing] | -26.442 | 11.666 | -2.267 | 0.024 |
|  | Wildebeest [Control] - Wildebeest [Ambush] | -12.278 | 6.391 | -1.921 | 0.055 |
|  | Wildebeest [Control] - Wildebeest [Coursing] | 0.506 | 9.627 | 0.053 | 0.958 |
|  | Wildebeest [Control] - Zebra [Control] | -35.047 | 10.565 | -3.317 | 0.001 |
|  | Wildebeest [Control] - Zebra [Ambush] | -12.129 | 5.378 | -2.255 | 0.024 |
|  | Wildebeest [Control] - Zebra [Coursing] | -35.295 | 8.774 | -4.023 | 0.000 |
|  | Wildebeest [Ambush] - Wildebeest [Coursing] | 12.783 | 4.981 | 2.566 | 0.010 |
|  | Wildebeest [Ambush] - Zebra [Control] | -22.770 | 8.580 | -2.654 | 0.008 |
|  | Wildebeest [Ambush] - Zebra [Ambush] | 0.149 | 2.295 | 0.065 | 0.948 |
|  | Wildebeest [Ambush] - Zebra [Coursing] | -23.018 | 6.249 | -3.683 | 0.000 |
|  | Wildebeest [Coursing] - Zebra [Control] | -35.553 | 10.236 | -3.473 | 0.001 |
|  | Wildebeest [Coursing] - Zebra [Ambush] | -12.635 | 5.310 | -2.380 | 0.018 |
|  | Wildebeest [Coursing] - Zebra [Coursing] | -35.801 | 8.382 | -4.271 | 0.000 |
|  | Zebra [Control] - Zebra [Ambush] | 22.918 | 9.317 | 2.460 | 0.014 |
|  | Zebra [Control] - Zebra [Coursing] | -0.248 | 3.443 | -0.072 | 0.943 |
|  | Zebra [Ambush] - Zebra [Coursing] | -23.166 | 6.894 | -3.361 | 0.001 |
| Probability of alarm calling | Impala [Control] - Impala [Ambush] | 4.058 | 14.067 | 0.288 | 0.773 |
|  | Impala [Control] - Impala [Coursing] | 6.452 | 22.738 | 0.284 | 0.777 |
|  | Impala [Control] - Wildebeest [Control] | -34.576 | 12.503 | -2.766 | 0.006 |
|  | Impala [Control] - Wildebeest [Ambush] | -15.938 | 11.037 | -1.444 | 0.149 |
|  | Impala [Control] - Wildebeest [Coursing] | 13.190 | 12.281 | 1.074 | 0.283 |
|  | Impala [Control] - Zebra [Control] | 1.847 | 16.905 | 0.109 | 0.913 |
|  | Impala [Control] - Zebra [Ambush] | 2.915 | 11.137 | 0.262 | 0.794 |
|  | Impala [Control] - Zebra [Coursing] | 2.097 | 14.172 | 0.148 | 0.882 |
|  | Impala [Ambush] - Impala [Coursing] | 2.394 | 9.820 | 0.244 | 0.807 |
|  | Impala [Ambush] - Wildebeest [Control] | -38.634 | 6.822 | -5.663 | 0.000 |
|  | Impala [Ambush] - Wildebeest [Ambush] | -19.996 | 3.941 | -5.073 | 0.000 |
|  | Impala [Ambush] - Wildebeest [Coursing] | 9.132 | 6.902 | 1.323 | 0.186 |
|  | Impala [Ambush] - Zebra [Control] | -2.210 | 13.432 | -0.165 | 0.869 |
|  | Impala [Ambush] - Zebra [Ambush] | -1.143 | 4.232 | -0.270 | 0.787 |
|  | Impala [Ambush] - Zebra [Coursing] | -1.961 | 9.833 | -0.199 | 0.842 |
|  | Impala [Coursing] - Wildebeest [Control] | -41.028 | 13.372 | -3.068 | 0.002 |
|  | Impala [Coursing] - Wildebeest [Ambush] | -22.390 | 12.280 | -1.823 | 0.069 |
|  | Impala [Coursing] - Wildebeest [Coursing] | 6.738 | 13.620 | 0.495 | 0.621 |
|  | Impala [Coursing] - Zebra [Control] | -4.605 | 17.781 | -0.259 | 0.796 |
|  | Impala [Coursing] - Zebra [Ambush] | -3.537 | 12.371 | -0.286 | 0.775 |
|  | Impala [Coursing] - Zebra [Coursing] | -4.355 | 15.286 | -0.285 | 0.776 |
|  | Wildebeest [Control] - Wildebeest [Ambush] | 18.638 | 6.137 | 3.037 | 0.002 |
|  | Wildebeest [Control] - Wildebeest [Coursing] | 47.766 | 11.285 | 4.233 | 0.000 |
|  | Wildebeest [Control] - Zebra [Control] | 36.423 | 14.246 | 2.557 | 0.011 |
|  | Wildebeest [Control] - Zebra [Ambush] | 37.491 | 6.295 | 5.955 | 0.000 |
|  | Wildebeest [Control] - Zebra [Coursing] | 36.673 | 10.997 | 3.335 | 0.001 |
|  | Wildebeest [Ambush] - Wildebeest [Coursing] | 29.128 | 5.843 | 4.985 | 0.000 |
|  | Wildebeest [Ambush] - Zebra [Control] | 17.785 | 13.060 | 1.362 | 0.174 |
|  | Wildebeest [Ambush] - Zebra [Ambush] | 18.853 | 2.838 | 6.644 | 0.000 |
|  | Wildebeest [Ambush] - Zebra [Coursing] | 18.035 | 9.298 | 1.940 | 0.053 |
|  | Wildebeest [Coursing] - Zebra [Control] | -11.343 | 14.192 | -0.799 | 0.424 |
|  | Wildebeest [Coursing] - Zebra [Ambush] | -10.275 | 6.301 | -1.631 | 0.103 |
|  | Wildebeest [Coursing] - Zebra [Coursing] | -11.093 | 10.743 | -1.033 | 0.302 |
|  | Zebra [Control] - Zebra [Ambush] | 1.067 | 14.164 | 0.075 | 0.940 |
|  | Zebra [Control] - Zebra [Coursing] | 0.250 | 5.518 | 0.045 | 0.964 |
|  | Zebra [Ambush] - Zebra [Coursing] | -0.818 | 10.187 | -0.080 | 0.936 |
| Probability of clumping | Wildebeest [Control] - Wildebeest [Ambush] | 1.920 | 2.627 | 0.731 | 0.466 |
|  | Wildebeest [Control] - Wildebeest [Coursing] | 4.237 | 4.435 | 0.955 | 0.341 |
|  | Wildebeest [Control] - Zebra [Control] | 2.111 | 71669.762 | 0.000 | 1.000 |
|  | Wildebeest [Control] - Zebra [Ambush] | 1.417 | 16028.691 | 0.000 | 1.000 |
|  | Wildebeest [Control] - Zebra [Coursing] | 21.042 | 45880.183 | 0.000 | 1.000 |
|  | Wildebeest [Ambush] - Wildebeest [Coursing] | 2.317 | 2.232 | 1.038 | 0.301 |
|  | Wildebeest [Ambush] - Zebra [Control] | 0.190 | 71669.763 | 0.000 | 1.000 |
|  | Wildebeest [Ambush] - Zebra [Ambush] | -0.503 | 16028.691 | 0.000 | 1.000 |
|  | Wildebeest [Ambush] - Zebra [Coursing] | 19.122 | 45880.183 | 0.000 | 1.000 |
|  | Wildebeest [Coursing] - Zebra [Control] | -2.126 | 71669.763 | 0.000 | 1.000 |
|  | Wildebeest [Coursing] - Zebra [Ambush] | -2.820 | 16028.691 | 0.000 | 1.000 |
|  | Wildebeest [Coursing] - Zebra [Coursing] | 16.805 | 45880.183 | 0.000 | 1.000 |
|  | Zebra [Control] - Zebra [Ambush] | -0.694 | 87698.454 | 0.000 | 1.000 |
|  | Zebra [Control] - Zebra [Coursing] | 18.932 | 30743.999 | 0.001 | 1.000 |
|  | Zebra [Ambush] - Zebra [Coursing] | 19.625 | 61400.873 | 0.000 | 1.000 |
