# Supplementary material for: Reactive anti-predator behavioral strategy shaped by predator characteristics
Source: PLoS One. 2021 Aug 18;16(8):e0256147. doi: 10.1371/journal.pone.0256147 (PMC8372962; doi:10.1371/journal.pone.0256147)
Supplement: S7 Table — GLMM results for intensity of prey anti-predator response (vigilance duration, alarm call frequency, latency to flee, and latency to alarm call) during encounters with predator models. Note that the interaction effect tests the difference between simple slopes (continuous) or effects (categorial), not whether each simple slope/effect is different from 0. Post hoc tests (see S7 and S8 Tables) are used to evaluate differences between and support for interacting variables. For these models, the reference level for prey species in impala, for habitat is open habitat, and for hunting strategy is the control model. (DOCX) [file pone.0256147.s008.docx]

**“Reactive anti-predator behavioral strategy shaped by predator characteristics”**

**S7 Table. Intensity of response.** GLMM results for intensity of prey anti-predator response (vigilance duration, alarm call frequency, latency to flee, and latency to alarm call) during encounters with predator models. Note that the interaction effect tests the difference between simple slopes (continuous) or effects (categorial), not whether each simple slope/effect is different from 0. Post hoc tests (see Tables S7, S8) are used to evaluate differences between and support for interacting variables. For these models, the reference level for prey species in impala, for habitat is open habitat, and for hunting strategy is the control model.

|  | Coefficient | Estimate (slope or HR) | SE | z value | p value |
| --- | --- | --- | --- | --- | --- |
| Duration of vigilance | Strategy [ambush] | -1.742 | 0.828 | -2.11 | 0.035 |
|  | Strategy [coursing] | -3.100 | 1.318 | -2.35 | 0.019 |
|  | Prey [wildebeest] | -0.718 | 0.841 | -0.85 | 0.393 |
|  | Prey [zebra] | -0.477 | 0.912 | -0.52 | 0.601 |
|  | Density | 0.086 | 0.147 | 0.58 | 0.560 |
|  | Preference | 0.342 | 0.333 | 1.03 | 0.304 |
|  | Success | 0.979 | 0.336 | 2.91 | 0.004 |
|  | Strategy [ambush] x Prey [wildebeest] | 1.206 | 0.901 | 1.34 | 0.181 |
|  | Strategy [coursing] x Prey [wildebeest] | 2.136 | 1.455 | 1.47 | 0.142 |
|  | Strategy [ambush] x Prey [zebra] | 0.752 | 1.058 | 0.71 | 0.477 |
|  | Strategy [coursing] x Prey [zebra] | 2.990 | 1.341 | 2.23 | 0.026 |
|  | Density x Prey [wildebeest] | 0.022 | 0.185 | 0.12 | 0.906 |
|  | Density x Prey [zebra] | 0.019 | 0.224 | 0.09 | 0.931 |
|  | Preference x Prey [wildebeest] | -0.099 | 0.404 | -0.24 | 0.807 |
|  | Preference x Prey [zebra] | -0.302 | 0.434 | -0.70 | 0.487 |
|  | Success x Prey [wildebeest] | -0.722 | 0.395 | -1.83 | 0.067 |
|  | Success x Prey [zebra | -0.410 | 0.582 | -0.70 | 0.481 |
| Frequency of alarm calling | Strategy [ambush] | -7.137 | 4.331 | -1.65 | 0.099 |
|  | Strategy [coursing] | -12.285 | 7.036 | -1.75 | 0.081 |
|  | Prey [wildebeest] | -3.356 | 4.218 | -0.80 | 0.426 |
|  | Prey [zebra] | -5.646 | 5.154 | -1.10 | 0.273 |
|  | Density | 1.173 | 0.687 | 1.71 | 0.088 |
|  | Preference | 3.575 | 1.581 | 2.26 | 0.024 |
|  | Success | 2.353 | 1.928 | 1.22 | 0.222 |
|  | Herd size | 0.521 | 0.176 | 2.97 | 0.003 |
|  | Mixed species [present] | -1.000 | 0.339 | -2.95 | 0.003 |
|  | Strategy [ambush] x Prey [wildebeest] | 7.057 | 4.626 | 1.53 | 0.127 |
|  | Strategy [coursing] x Prey [wildebeest] | 9.404 | 7.572 | 1.24 | 0.214 |
|  | Strategy [ambush] x Prey [zebra] | 4.545 | 6.026 | 0.75 | 0.451 |
|  | Strategy [coursing] x Prey [zebra] | 11.855 | 7.235 | 1.64 | 0.101 |
|  | Density x Prey [wildebeest] | -1.760 | 0.842 | -2.09 | 0.037 |
|  | Density x Prey [zebra] | -0.376 | 1.159 | -0.32 | 0.746 |
|  | Preference x Prey [wildebeest] | -2.219 | 1.877 | -1.18 | 0.237 |
|  | Preference x Prey [zebra] | -4.310 | 2.156 | -2.00 | 0.046 |
|  | Success x Prey [wildebeest] | -0.959 | 2.146 | -0.45 | 0.655 |
|  | Success x Prey [zebra | 0.363 | 3.573 | 0.10 | 0.919 |
| Latency to flee | Strategy [ambush] | 1.573 | 2.441 | 0.19 | 0.853 |
|  | Strategy [coursing] | 0.191 | 3.910 | -0.42 | 0.673 |
|  | Prey [wildebeest] | 0.018 | 2.702 | -1.49 | 0.136 |
|  | Prey [zebra] | 48.143 | 3.124 | 1.24 | 0.215 |
|  | Density | 0.668 | 0.439 | -0.92 | 0.359 |
|  | Preference | 3.408 | 1.018 | 1.20 | 0.228 |
|  | Success | 0.759 | 0.961 | -0.29 | 0.775 |
|  | Habitat [closed] | 3.594 | 0.245 | 5.22 | 0.000 |
|  | Herd size | 0.859 | 0.128 | -1.19 | 0.233 |
|  | Juveniles [present] | 1.359 | 0.370 | 0.83 | 0.406 |
|  | Strategy [ambush] x Prey [wildebeest] | 10.051 | 2.819 | 0.82 | 0.413 |
|  | Strategy [coursing] x Prey [wildebeest] | 208.255 | 4.595 | 1.16 | 0.245 |
|  | Strategy [ambush] x Prey [zebra] | 0.002 | 3.627 | -1.75 | 0.080 |
|  | Strategy [coursing] x Prey [zebra] | 2.694 | 4.031 | 0.25 | 0.806 |
|  | Density x Prey [wildebeest] | 1.239 | 0.619 | 0.35 | 0.729 |
|  | Density x Prey [zebra] | 1.450 | 0.744 | 0.50 | 0.617 |
|  | Preference x Prey [wildebeest] | 0.101 | 1.308 | -1.75 | 0.080 |
|  | Preference x Prey [zebra] | 77.928 | 2.186 | 1.99 | 0.046 |
|  | Success x Prey [wildebeest] | 2.059 | 1.317 | 0.55 | 0.583 |
|  | Success x Prey [zebra] | 0.898 | 1.381 | -0.08 | 0.938 |
| Latency to alarm call | Strategy [ambush] | 0.007 | 5.775 | -0.85 | 0.395 |
|  | Strategy [coursing] | 0.000 | 9.362 | -1.00 | 0.315 |
|  | Prey [wildebeest] | 0.003 | 5.648 | -1.04 | 0.296 |
|  | Prey [zebra] | 0.011 | 6.333 | -0.72 | 0.472 |
|  | Density | 2.190 | 0.832 | 0.94 | 0.346 |
|  | Preference | 4.101 | 2.598 | 0.54 | 0.587 |
|  | Success | 61.162 | 2.151 | 1.91 | 0.056 |
|  | Strategy [ambush] x Prey [wildebeest] | 1099.040 | 6.147 | 1.14 | 0.255 |
|  | Strategy [coursing] x Prey [wildebeest] | 80830.833 | 10.092 | 1.12 | 0.263 |
|  | Strategy [ambush] x Prey [zebra] | 2.450 | 7.369 | 0.12 | 0.903 |
|  | Strategy [coursing] x Prey [zebra] | 3367.128 | 9.486 | 0.86 | 0.392 |
|  | Density x Prey [wildebeest] | 0.360 | 1.020 | -1.00 | 0.316 |
|  | Density x Prey [zebra] | 1.909 | 1.382 | 0.47 | 0.640 |
|  | Preference x Prey [wildebeest] | 0.145 | 2.896 | -0.67 | 0.504 |
|  | Preference x Prey [zebra] | 10.376 | 4.264 | 0.55 | 0.583 |
|  | Success x Prey [wildebeest] | 0.016 | 2.547 | -1.63 | 0.102 |
|  | Success x Prey [zebra] | 0.002 | 2.802 | -2.15 | 0.031 |
