# Supplementary material for: Reactive anti-predator behavioral strategy shaped by predator characteristics
Source: PLoS One. 2021 Aug 18;16(8):e0256147. doi: 10.1371/journal.pone.0256147 (PMC8372962; doi:10.1371/journal.pone.0256147)
Supplement: S8 Table — Post-hoc interaction analysis of GLMM results from S6 Table using package ‘emmeans’ [83]. (A) Simple slopes (estimates of slopes of the covariate trend of each level of the factor; continuous covariates) and (B) simple effects (general contrasts of factor levels; categorical covariates) are presented for each prey species. (DOCX) [file pone.0256147.s009.docx]

**“Reactive anti-predator behavioral strategy shaped by predator characteristics”**

**S8 Table. Intensity of response.** Post-hoc interaction analysis of GLMM results from Table S6 using package ‘emmeans’ [83]. (A) Simple slopes (estimates of slopes of the covariate trend of each level of the factor; continuous covariates) and (B) simple effects (general contrasts of factor levels; categorical covariates) are presented for each prey species.

(A) Simply slopes

|  | Predictor | Species | Simple slopes | SE | 95% CI* |
| --- | --- | --- | --- | --- | --- |
| Duration of vigilance | Density | Impala | 0.086 | 0.147 | (-0.204, 0.375) |
|  |  | Wildebeest | 0.150 | 0.108 | (-0.061, 0.361) |
|  |  | Zebra | 0.105 | 0.169 | (-0.226, 0.437) |
|  | Preference | Impala | -0.364 | 0.243 | (-0.841, 0.112) |
|  |  | Wildebeest | -0.125 | 0.206 | (-0.528, 0.279) |
|  |  | Zebra | 0.373 | 0.278 | (-0.174, 0.919) |
|  | Success | Impala | 0.180 | 0.096 | (-0.007, 0.368) |
|  |  | Wildebeest | -0.115 | 0.131 | (-0.372, 0.142) |
|  |  | Zebra | -0.389 | 0.169 | (-0.721, -0.057) |
| Frequency of alarm calling | Density | Impala | 1.173 | 0.687 | (-0.176, 2.521) |
|  |  | Wildebeest | -0.587 | 0.486 | (-1.541, 0.368) |
|  |  | Zebra | 0.797 | 0.931 | (-1.030, 2.624) |
|  | Preference | Impala | 0.895 | 1.236 | (-1.531, 3.322) |
|  |  | Wildebeest | -0.875 | 0.907 | (-2.655, 0.906) |
|  |  | Zebra | 0.083 | 1.629 | (-3.115, 3.281) |
|  | Success | Impala | -0.677 | 0.523 | (-1.704, 0.350) |
|  |  | Wildebeest | -1.190 | 0.598 | (-2.363, -0.017) |
|  |  | Zebra | 0.362 | 1.022 | (-1.644, 2.368) |
| Latency to flee | Density | Impala | -0.414 | 0.440 | (-1.275, 0.448) |
|  |  | Wildebeest | -0.155 | 0.437 | (-1.012, 0.702) |
|  |  | Zebra | 0.050 | 0.614 | (-1.153, 1.253) |
|  | Preference | Impala | -1.350 | 0.747 | (-2.814, 0.113) |
|  |  | Wildebeest | 1.210 | 0.752 | (-0.263, 2.683) |
|  |  | Zebra | 1.441 | 1.067 | (-0.650, 3.532) |
|  | Success | Impala | -0.072 | 0.288 | (-0.636, 0.492) |
|  |  | Wildebeest | -0.144 | 0.508 | (-1.140, 0.851) |
|  |  | Zebra | 0.018 | 0.653 | (-1.262, 1.299) |
| Latency to alarm | Density | Impala | 0.390 | 0.716 | (-1.012, 1.793) |
|  |  | Wildebeest | -0.366 | 0.533 | (-1.410, 0.679) |
|  |  | Zebra | 1.017 | 0.936 | (-0.817, 2.851) |
|  | Preference | Impala | 0.944 | 1.366 | (-1.734, 3.622) |
|  |  | Wildebeest | -0.157 | 0.941 | (-2.001, 1.688) |
|  |  | Zebra | -0.589 | 1.655 | (-3.833, 2.654) |
|  | Success | Impala | -0.942 | 0.574 | (-2.068, 0.184) |
|  |  | Wildebeest | -0.765 | 0.617 | (-1.975, 0.445) |
|  |  | Zebra | 0.856 | 1.023 | (-1.149, 2.860) |

(B) Simple effects (hunting style)

|  | Level | Species | Estimate | SE | t/z ratio | p value |
| --- | --- | --- | --- | --- | --- | --- |
| Duration of vigilance | Control | Impala | -0.060 | 0.843 | -0.071 | 0.943 |
|  |  | Wildebeest | -0.766 | 0.957 | -0.800 | 0.635 |
|  |  | Zebra | 0.826 | 0.760 | 1.087 | 0.635 |
|  | Ambush | Impala | 2.030 | 0.519 | 3.915 | 0.000 |
|  |  | Wildebeest | -0.997 | 0.647 | -1.541 | 0.123 |
|  |  | Zebra | -1.033 | 0.580 | -1.781 | 0.112 |
|  | Coursing | Impala | 1.546 | 0.404 | 3.826 | 0.000 |
|  |  | Wildebeest | -1.351 | 0.321 | -4.202 | 0.000 |
|  |  | Zebra | -0.195 | 0.344 | -0.567 | 0.570 |
| Frequency of alarm calling | Control | Impala | -0.400 | 1.293 | -0.309 | 0.957 |
|  |  | Wildebeest | 0.067 | 1.237 | 0.054 | 0.957 |
|  |  | Zebra | 0.333 | 1.139 | 0.293 | 0.957 |
|  | Ambush | Impala | -0.521 | 0.829 | -0.629 | 0.530 |
|  |  | Wildebeest | 2.086 | 0.849 | 2.455 | 0.042 |
|  |  | Zebra | -1.564 | 0.870 | -1.799 | 0.108 |
|  | Coursing | Impala | 0.065 | 0.666 | 0.098 | 0.922 |
|  |  | Wildebeest | 1.172 | 0.461 | 2.542 | 0.032 |
|  |  | Zebra | -1.238 | 0.538 | -2.300 | 0.032 |
| Latency to flee | Control | Impala | 0.003 | 0.250 | 0.010 | 0.992 |
|  |  | Wildebeest | 0.012 | 0.251 | 0.047 | 0.992 |
|  |  | Zebra | -0.014 | 0.202 | -0.071 | 0.992 |
|  | Ambush | Impala | -0.234 | 0.156 | -1.499 | 0.201 |
|  |  | Wildebeest | 0.363 | 0.172 | 2.112 | 0.105 |
|  |  | Zebra | -0.129 | 0.156 | -0.826 | 0.409 |
|  | Coursing | Impala | -0.013 | 0.131 | -0.100 | 0.921 |
|  |  | Wildebeest | -0.032 | 0.093 | -0.342 | 0.921 |
|  |  | Zebra | 0.045 | 0.099 | 0.451 | 0.921 |
| Latency to alarm | Control | Impala | 0.943 | 1.239 | 0.761 | 0.670 |
|  |  | Wildebeest | -0.950 | 1.191 | -0.798 | 0.670 |
|  |  | Zebra | 0.008 | 1.119 | 0.007 | 0.995 |
|  | Ambush | Impala | -1.945 | 0.802 | -2.424 | 0.023 |
|  |  | Wildebeest | 3.788 | 0.848 | 4.469 | 0.000 |
|  |  | Zebra | -1.843 | 0.869 | -2.120 | 0.034 |
|  | Coursing | Impala | -0.637 | 0.654 | -0.974 | 0.330 |
|  |  | Wildebeest | 1.788 | 0.454 | 3.938 | 0.000 |
|  |  | Zebra | -1.151 | 0.537 | -2.145 | 0.048 |
