# Supplementary material for: Reactive anti-predator behavioral strategy shaped by predator characteristics
Source: PLoS One. 2021 Aug 18;16(8):e0256147. doi: 10.1371/journal.pone.0256147 (PMC8372962; doi:10.1371/journal.pone.0256147)
Supplement: S9 Table — Post-hoc interaction analysis of GLMM results from S6 Table using package ‘emmeans’ [83], evaluating the pairwise differences between species responses (i.e., conditional contrasts) for (A) continuous and (B) categorical predictors. (DOCX) [file pone.0256147.s010.docx]

**“Reactive anti-predator behavioral strategy shaped by predator characteristics”**

**S9 Table. Intensity of response.** Post-hoc interaction analysis of GLMM results from Table S6 using package ‘emmeans’ [83], evaluating the pairwise differences between species responses (i.e., conditional contrasts) for (A) continuous and (B) categorical predictors.

(A) Continuous predictors

|  | Predictor | Contrast | Estimate | SE | t/z ratio | p value |
| --- | --- | --- | --- | --- | --- | --- |
| Duration of vigilance | Density | Impala-Wildebeest | -0.064 | 0.182 | -0.353 | 0.934 |
|  |  | Impala-Zebra | -0.020 | 0.224 | -0.088 | 0.996 |
|  |  | Wildebeest-Zebra | 0.045 | 0.200 | 0.223 | 0.973 |
|  | Preference | Impala-Wildebeest | -0.240 | 0.318 | -0.754 | 0.731 |
|  |  | Impala-Zebra | -0.737 | 0.369 | -1.997 | 0.114 |
|  |  | Wildebeest-Zebra | -0.498 | 0.346 | -1.437 | 0.322 |
|  | Success | Impala-Wildebeest | 0.295 | 0.162 | 1.821 | 0.163 |
|  |  | Impala-Zebra | 0.569 | 0.194 | 2.929 | 0.010 |
|  |  | Wildebeest-Zebra | 0.274 | 0.214 | 1.278 | 0.408 |
| Frequency of alarm calling | Density | Impala-Wildebeest | 1.760 | 0.842 | 2.089 | 0.093 |
|  |  | Impala-Zebra | 0.376 | 1.159 | 0.324 | 0.944 |
|  |  | Wildebeest-Zebra | -1.384 | 1.051 | -1.317 | 0.386 |
|  | Preference | Impala-Wildebeest | 1.770 | 1.535 | 1.153 | 0.482 |
|  |  | Impala-Zebra | 0.813 | 2.047 | 0.397 | 0.917 |
|  |  | Wildebeest-Zebra | -0.958 | 1.868 | -0.513 | 0.865 |
|  | Success | Impala-Wildebeest | 0.514 | 0.802 | 0.640 | 0.798 |
|  |  | Impala-Zebra | -1.039 | 1.162 | -0.894 | 0.644 |
|  |  | Wildebeest-Zebra | -1.553 | 1.173 | -1.323 | 0.382 |
| Latency to flee | Density | Impala-Wildebeest | -0.259 | 0.619 | -0.418 | 0.908 |
|  |  | Impala-Zebra | -0.463 | 0.749 | -0.618 | 0.810 |
|  |  | Wildebeest-Zebra | -0.205 | 0.753 | -0.272 | 0.960 |
|  | Preference | Impala-Wildebeest | -2.560 | 1.063 | -2.409 | 0.042 |
|  |  | Impala-Zebra | -2.791 | 1.320 | -2.114 | 0.087 |
|  |  | Wildebeest-Zebra | -0.231 | 1.300 | -0.178 | 0.983 |
|  | Success | Impala-Wildebeest | 0.072 | 0.589 | 0.123 | 0.992 |
|  |  | Impala-Zebra | -0.090 | 0.723 | -0.125 | 0.991 |
|  |  | Wildebeest-Zebra | -0.163 | 0.822 | -0.198 | 0.979 |
| Latency to alarm | Density | Impala-Wildebeest | 0.756 | 0.873 | 0.866 | 0.662 |
|  |  | Impala-Zebra | -0.627 | 1.163 | -0.539 | 0.852 |
|  |  | Wildebeest-Zebra | -1.383 | 1.072 | -1.289 | 0.401 |
|  | Preference | Impala-Wildebeest | 1.101 | 1.673 | 0.658 | 0.788 |
|  |  | Impala-Zebra | 1.533 | 2.172 | 0.706 | 0.760 |
|  |  | Wildebeest-Zebra | 0.433 | 1.890 | 0.229 | 0.972 |
|  | Success | Impala-Wildebeest | -0.177 | 0.846 | -0.209 | 0.976 |
|  |  | Impala-Zebra | -1.798 | 1.186 | -1.516 | 0.283 |
|  |  | Wildebeest-Zebra | -1.621 | 1.185 | -1.368 | 0.358 |

(B) Categorical predictors

|  | Contrast | Estimate | SE | t/z ratio | p value |
| --- | --- | --- | --- | --- | --- |
| Duration of vigilance | Impala [Control] - Impala [Ambush] | 1.723 | 0.821 | 2.100 | 0.036 |
|  | Impala [Control] - Impala [Coursing] | 3.096 | 1.304 | 2.375 | 0.018 |
|  | Impala [Control] - Wildebeest [Control] | 0.720 | 0.715 | 1.007 | 0.314 |
|  | Impala [Control] - Wildebeest [Ambush] | 1.250 | 0.644 | 1.943 | 0.052 |
|  | Impala [Control] - Wildebeest [Coursing] | 1.670 | 0.730 | 2.287 | 0.022 |
|  | Impala [Control] - Zebra [Control] | 0.354 | 0.869 | 0.407 | 0.684 |
|  | Impala [Control] - Zebra [Ambush] | 1.344 | 0.647 | 2.076 | 0.038 |
|  | Impala [Control] - Zebra [Coursing] | 0.464 | 0.764 | 0.607 | 0.544 |
|  | Impala [Ambush] - Impala [Coursing] | 1.373 | 0.547 | 2.509 | 0.012 |
|  | Impala [Ambush] - Wildebeest [Control] | -1.003 | 0.385 | -2.607 | 0.009 |
|  | Impala [Ambush] - Wildebeest [Ambush] | -0.473 | 0.226 | -2.091 | 0.037 |
|  | Impala [Ambush] - Wildebeest [Coursing] | -0.053 | 0.413 | -0.128 | 0.898 |
|  | Impala [Ambush] - Zebra [Control] | -1.369 | 0.627 | -2.184 | 0.029 |
|  | Impala [Ambush] - Zebra [Ambush] | -0.379 | 0.237 | -1.599 | 0.110 |
|  | Impala [Ambush] - Zebra [Coursing] | -1.260 | 0.469 | -2.684 | 0.007 |
|  | Impala [Coursing] - Wildebeest [Control] | -2.376 | 0.758 | -3.136 | 0.002 |
|  | Impala [Coursing] - Wildebeest [Ambush] | -1.846 | 0.691 | -2.672 | 0.008 |
|  | Impala [Coursing] - Wildebeest [Coursing] | -1.426 | 0.772 | -1.846 | 0.065 |
|  | Impala [Coursing] - Zebra [Control] | -2.742 | 0.905 | -3.030 | 0.003 |
|  | Impala [Coursing] - Zebra [Ambush] | -1.752 | 0.695 | -2.523 | 0.012 |
|  | Impala [Coursing] - Zebra [Coursing] | -2.633 | 0.804 | -3.274 | 0.001 |
|  | Wildebeest [Control] - Wildebeest [Ambush] | 0.530 | 0.369 | 1.437 | 0.151 |
|  | Wildebeest [Control] - Wildebeest [Coursing] | 0.950 | 0.658 | 1.443 | 0.149 |
|  | Wildebeest [Control] - Zebra [Control] | -0.366 | 0.675 | -0.542 | 0.588 |
|  | Wildebeest [Control] - Zebra [Ambush] | 0.624 | 0.344 | 1.812 | 0.070 |
|  | Wildebeest [Control] - Zebra [Coursing] | -0.256 | 0.532 | -0.482 | 0.630 |
|  | Wildebeest [Ambush] - Wildebeest [Coursing] | 0.420 | 0.345 | 1.216 | 0.224 |
|  | Wildebeest [Ambush] - Zebra [Control] | -0.896 | 0.599 | -1.496 | 0.135 |
|  | Wildebeest [Ambush] - Zebra [Ambush] | 0.094 | 0.148 | 0.637 | 0.524 |
|  | Wildebeest [Ambush] - Zebra [Coursing] | -0.787 | 0.431 | -1.825 | 0.068 |
|  | Wildebeest [Coursing] - Zebra [Control] | -1.316 | 0.691 | -1.904 | 0.057 |
|  | Wildebeest [Coursing] - Zebra [Ambush] | -0.326 | 0.375 | -0.869 | 0.385 |
|  | Wildebeest [Coursing] - Zebra [Coursing] | -1.207 | 0.552 | -2.185 | 0.029 |
|  | Zebra [Control] - Zebra [Ambush] | 0.990 | 0.650 | 1.524 | 0.128 |
|  | Zebra [Control] - Zebra [Coursing] | 0.109 | 0.244 | 0.449 | 0.653 |
|  | Zebra [Ambush] - Zebra [Coursing] | -0.881 | 0.470 | -1.873 | 0.061 |
|  | Impala [Control] - Impala [Ambush] | 7.032 | 4.390 | 1.602 | 0.110 |
|  | Impala [Control] - Impala [Coursing] | 12.239 | 7.140 | 1.714 | 0.087 |
|  | Impala [Control] - Wildebeest [Control] | 1.397 | 3.804 | 0.367 | 0.714 |
|  | Impala [Control] - Wildebeest [Ambush] | 2.806 | 3.443 | 0.815 | 0.415 |
|  | Impala [Control] - Wildebeest [Coursing] | 7.334 | 3.893 | 1.884 | 0.060 |
|  | Impala [Control] - Zebra [Control] | 3.531 | 5.191 | 0.680 | 0.496 |
|  | Impala [Control] - Zebra [Ambush] | 6.200 | 3.496 | 1.773 | 0.076 |
|  | Impala [Control] - Zebra [Coursing] | 4.037 | 4.374 | 0.923 | 0.356 |
|  | Impala [Ambush] - Impala [Coursing] | 5.207 | 3.032 | 1.717 | 0.086 |
|  | Impala [Ambush] - Wildebeest [Control] | -5.635 | 1.971 | -2.858 | 0.004 |
|  | Impala [Ambush] - Wildebeest [Ambush] | -4.226 | 1.172 | -3.606 | 0.000 |
|  | Impala [Ambush] - Wildebeest [Coursing] | 0.302 | 2.152 | 0.140 | 0.888 |
|  | Impala [Ambush] - Zebra [Control] | -3.500 | 4.061 | -0.862 | 0.389 |
|  | Impala [Ambush] - Zebra [Ambush] | -0.832 | 1.284 | -0.648 | 0.517 |
|  | Impala [Ambush] - Zebra [Coursing] | -2.994 | 2.976 | -1.006 | 0.315 |
|  | Impala [Coursing] - Wildebeest [Control] | -10.842 | 4.147 | -2.614 | 0.009 |
|  | Impala [Coursing] - Wildebeest [Ambush] | -9.433 | 3.836 | -2.459 | 0.014 |
|  | Impala [Coursing] - Wildebeest [Coursing] | -4.905 | 4.233 | -1.159 | 0.247 |
|  | Impala [Coursing] - Zebra [Control] | -8.707 | 5.465 | -1.593 | 0.111 |
|  | Impala [Coursing] - Zebra [Ambush] | -6.039 | 3.865 | -1.563 | 0.119 |
|  | Impala [Coursing] - Zebra [Coursing] | -8.202 | 4.724 | -1.736 | 0.083 |
|  | Wildebeest [Control] - Wildebeest [Ambush] | 1.409 | 1.821 | 0.774 | 0.439 |
|  | Wildebeest [Control] - Wildebeest [Coursing] | 5.937 | 3.399 | 1.747 | 0.081 |
|  | Wildebeest [Control] - Zebra [Control] | 2.135 | 4.218 | 0.506 | 0.613 |
|  | Wildebeest [Control] - Zebra [Ambush] | 4.803 | 1.779 | 2.700 | 0.007 |
|  | Wildebeest [Control] - Zebra [Coursing] | 2.641 | 3.189 | 0.828 | 0.408 |
|  | Wildebeest [Ambush] - Wildebeest [Coursing] | 4.528 | 1.811 | 2.501 | 0.013 |
|  | Wildebeest [Ambush] - Zebra [Control] | 0.726 | 3.923 | 0.185 | 0.853 |
|  | Wildebeest [Ambush] - Zebra [Ambush] | 3.394 | 0.815 | 4.167 | 0.000 |
|  | Wildebeest [Ambush] - Zebra [Coursing] | 1.231 | 2.778 | 0.443 | 0.658 |
|  | Wildebeest [Coursing] - Zebra [Control] | -3.802 | 4.341 | -0.876 | 0.381 |
|  | Wildebeest [Coursing] - Zebra [Ambush] | -1.134 | 1.991 | -0.569 | 0.569 |
|  | Wildebeest [Coursing] - Zebra [Coursing] | -3.297 | 3.334 | -0.989 | 0.323 |
|  | Zebra [Control] - Zebra [Ambush] | 2.669 | 4.263 | 0.626 | 0.532 |
|  | Zebra [Control] - Zebra [Coursing] | 0.506 | 1.637 | 0.309 | 0.757 |
|  | Zebra [Ambush] - Zebra [Coursing] | -2.163 | 3.060 | -0.707 | 0.480 |
| Frequency of alarm calling | Impala [Control] - Impala [Ambush] | 7.032 | 4.390 | 1.602 | 0.110 |
|  | Impala [Control] - Impala [Coursing] | 12.239 | 7.140 | 1.714 | 0.087 |
|  | Impala [Control] - Wildebeest [Control] | 1.397 | 3.804 | 0.367 | 0.714 |
|  | Impala [Control] - Wildebeest [Ambush] | 2.806 | 3.443 | 0.815 | 0.415 |
|  | Impala [Control] - Wildebeest [Coursing] | 7.334 | 3.893 | 1.884 | 0.060 |
|  | Impala [Control] - Zebra [Control] | 3.531 | 5.191 | 0.680 | 0.496 |
|  | Impala [Control] - Zebra [Ambush] | 6.200 | 3.496 | 1.773 | 0.076 |
|  | Impala [Control] - Zebra [Coursing] | 4.037 | 4.374 | 0.923 | 0.356 |
|  | Impala [Ambush] - Impala [Coursing] | 5.207 | 3.032 | 1.717 | 0.086 |
|  | Impala [Ambush] - Wildebeest [Control] | -5.635 | 1.971 | -2.858 | 0.004 |
|  | Impala [Ambush] - Wildebeest [Ambush] | -4.226 | 1.172 | -3.606 | 0.000 |
|  | Impala [Ambush] - Wildebeest [Coursing] | 0.302 | 2.152 | 0.140 | 0.888 |
|  | Impala [Ambush] - Zebra [Control] | -3.500 | 4.061 | -0.862 | 0.389 |
|  | Impala [Ambush] - Zebra [Ambush] | -0.832 | 1.284 | -0.648 | 0.517 |
|  | Impala [Ambush] - Zebra [Coursing] | -2.994 | 2.976 | -1.006 | 0.315 |
|  | Impala [Coursing] - Wildebeest [Control] | -10.842 | 4.147 | -2.614 | 0.009 |
|  | Impala [Coursing] - Wildebeest [Ambush] | -9.433 | 3.836 | -2.459 | 0.014 |
|  | Impala [Coursing] - Wildebeest [Coursing] | -4.905 | 4.233 | -1.159 | 0.247 |
|  | Impala [Coursing] - Zebra [Control] | -8.707 | 5.465 | -1.593 | 0.111 |
|  | Impala [Coursing] - Zebra [Ambush] | -6.039 | 3.865 | -1.563 | 0.119 |
|  | Impala [Coursing] - Zebra [Coursing] | -8.202 | 4.724 | -1.736 | 0.083 |
|  | Wildebeest [Control] - Wildebeest [Ambush] | 1.409 | 1.821 | 0.774 | 0.439 |
|  | Wildebeest [Control] - Wildebeest [Coursing] | 5.937 | 3.399 | 1.747 | 0.081 |
|  | Wildebeest [Control] - Zebra [Control] | 2.135 | 4.218 | 0.506 | 0.613 |
|  | Wildebeest [Control] - Zebra [Ambush] | 4.803 | 1.779 | 2.700 | 0.007 |
|  | Wildebeest [Control] - Zebra [Coursing] | 2.641 | 3.189 | 0.828 | 0.408 |
|  | Wildebeest [Ambush] - Wildebeest [Coursing] | 4.528 | 1.811 | 2.501 | 0.013 |
|  | Wildebeest [Ambush] - Zebra [Control] | 0.726 | 3.923 | 0.185 | 0.853 |
|  | Wildebeest [Ambush] - Zebra [Ambush] | 3.394 | 0.815 | 4.167 | 0.000 |
|  | Wildebeest [Ambush] - Zebra [Coursing] | 1.231 | 2.778 | 0.443 | 0.658 |
|  | Wildebeest [Coursing] - Zebra [Control] | -3.802 | 4.341 | -0.876 | 0.381 |
|  | Wildebeest [Coursing] - Zebra [Ambush] | -1.134 | 1.991 | -0.569 | 0.569 |
|  | Wildebeest [Coursing] - Zebra [Coursing] | -3.297 | 3.334 | -0.989 | 0.323 |
|  | Zebra [Control] - Zebra [Ambush] | 2.669 | 4.263 | 0.626 | 0.532 |
|  | Zebra [Control] - Zebra [Coursing] | 0.506 | 1.637 | 0.309 | 0.757 |
|  | Zebra [Ambush] - Zebra [Coursing] | -2.163 | 3.060 | -0.707 | 0.480 |
| Latency to flee | Impala [Control] - Impala [Ambush] | 0.024 | 2.463 | 0.010 | 0.992 |
|  | Impala [Control] - Impala [Coursing] | 2.243 | 3.940 | 0.569 | 0.569 |
|  | Impala [Control] - Wildebeest [Control] | 5.334 | 2.341 | 2.278 | 0.023 |
|  | Impala [Control] - Wildebeest [Ambush] | 1.989 | 1.971 | 1.009 | 0.313 |
|  | Impala [Control] - Wildebeest [Coursing] | 0.440 | 2.398 | 0.183 | 0.855 |
|  | Impala [Control] - Zebra [Control] | -4.280 | 3.148 | -1.360 | 0.174 |
|  | Impala [Control] - Zebra [Ambush] | 1.835 | 1.987 | 0.924 | 0.356 |
|  | Impala [Control] - Zebra [Coursing] | -3.548 | 2.613 | -1.358 | 0.174 |
|  | Impala [Ambush] - Impala [Coursing] | 2.219 | 1.680 | 1.321 | 0.186 |
|  | Impala [Ambush] - Wildebeest [Control] | 5.309 | 1.465 | 3.623 | 0.000 |
|  | Impala [Ambush] - Wildebeest [Ambush] | 1.965 | 0.706 | 2.781 | 0.005 |
|  | Impala [Ambush] - Wildebeest [Coursing] | 0.415 | 1.528 | 0.272 | 0.786 |
|  | Impala [Ambush] - Zebra [Control] | -4.304 | 2.556 | -1.684 | 0.092 |
|  | Impala [Ambush] - Zebra [Ambush] | 1.811 | 0.757 | 2.392 | 0.017 |
|  | Impala [Ambush] - Zebra [Coursing] | -3.572 | 1.845 | -1.936 | 0.053 |
|  | Impala [Coursing] - Wildebeest [Control] | 3.090 | 2.474 | 1.249 | 0.212 |
|  | Impala [Coursing] - Wildebeest [Ambush] | -0.255 | 2.110 | -0.121 | 0.904 |
|  | Impala [Coursing] - Wildebeest [Coursing] | -1.804 | 2.507 | -0.720 | 0.472 |
|  | Impala [Coursing] - Zebra [Control] | -6.523 | 3.246 | -2.010 | 0.044 |
|  | Impala [Coursing] - Zebra [Ambush] | -0.409 | 2.130 | -0.192 | 0.848 |
|  | Impala [Coursing] - Zebra [Coursing] | -5.791 | 2.717 | -2.131 | 0.033 |
|  | Wildebeest [Control] - Wildebeest [Ambush] | -3.345 | 1.500 | -2.229 | 0.026 |
|  | Wildebeest [Control] - Wildebeest [Coursing] | -4.894 | 2.626 | -1.864 | 0.062 |
|  | Wildebeest [Control] - Zebra [Control] | -9.613 | 2.815 | -3.415 | 0.001 |
|  | Wildebeest [Control] - Zebra [Ambush] | -3.499 | 1.403 | -2.494 | 0.013 |
|  | Wildebeest [Control] - Zebra [Coursing] | -8.881 | 2.197 | -4.043 | 0.000 |
|  | Wildebeest [Ambush] - Wildebeest [Coursing] | -1.549 | 1.351 | -1.147 | 0.252 |
|  | Wildebeest [Ambush] - Zebra [Control] | -6.268 | 2.509 | -2.498 | 0.012 |
|  | Wildebeest [Ambush] - Zebra [Ambush] | -0.154 | 0.567 | -0.271 | 0.786 |
|  | Wildebeest [Ambush] - Zebra [Coursing] | -5.537 | 1.781 | -3.109 | 0.002 |
|  | Wildebeest [Coursing] - Zebra [Control] | -4.719 | 2.853 | -1.654 | 0.098 |
|  | Wildebeest [Coursing] - Zebra [Ambush] | 1.395 | 1.464 | 0.953 | 0.341 |
|  | Wildebeest [Coursing] - Zebra [Coursing] | -3.987 | 2.238 | -1.782 | 0.075 |
|  | Zebra [Control] - Zebra [Ambush] | 6.114 | 2.712 | 2.255 | 0.024 |
|  | Zebra [Control] - Zebra [Coursing] | 0.732 | 1.025 | 0.714 | 0.475 |
|  | Zebra [Ambush] - Zebra [Coursing] | -5.383 | 1.945 | -2.767 | 0.006 |
| Latency to alarm call | Impala [Control] - Impala [Ambush] | 3.890 | 5.773 | 0.674 | 0.500 |
|  | Impala [Control] - Impala [Coursing] | 7.697 | 9.367 | 0.822 | 0.411 |
|  | Impala [Control] - Wildebeest [Control] | 2.361 | 4.993 | 0.473 | 0.636 |
|  | Impala [Control] - Wildebeest [Ambush] | 1.457 | 4.551 | 0.320 | 0.749 |
|  | Impala [Control] - Wildebeest [Coursing] | 3.888 | 4.963 | 0.783 | 0.433 |
|  | Impala [Control] - Zebra [Control] | 1.411 | 6.203 | 0.227 | 0.820 |
|  | Impala [Control] - Zebra [Ambush] | 4.713 | 4.597 | 1.025 | 0.305 |
|  | Impala [Control] - Zebra [Coursing] | 2.543 | 5.379 | 0.473 | 0.636 |
|  | Impala [Ambush] - Impala [Coursing] | 3.808 | 3.894 | 0.978 | 0.328 |
|  | Impala [Ambush] - Wildebeest [Control] | -1.528 | 2.353 | -0.649 | 0.516 |
|  | Impala [Ambush] - Wildebeest [Ambush] | -2.433 | 1.467 | -1.658 | 0.097 |
|  | Impala [Ambush] - Wildebeest [Coursing] | -0.002 | 2.690 | -0.001 | 0.999 |
|  | Impala [Ambush] - Zebra [Control] | -2.479 | 4.733 | -0.524 | 0.600 |
|  | Impala [Ambush] - Zebra [Ambush] | 0.824 | 1.596 | 0.516 | 0.606 |
|  | Impala [Ambush] - Zebra [Coursing] | -1.346 | 3.508 | -0.384 | 0.701 |
|  | Impala [Coursing] - Wildebeest [Control] | -5.336 | 5.235 | -1.019 | 0.308 |
|  | Impala [Coursing] - Wildebeest [Ambush] | -6.241 | 4.956 | -1.259 | 0.208 |
|  | Impala [Coursing] - Wildebeest [Coursing] | -3.810 | 5.512 | -0.691 | 0.489 |
|  | Impala [Coursing] - Zebra [Control] | -6.287 | 6.822 | -0.922 | 0.357 |
|  | Impala [Coursing] - Zebra [Ambush] | -2.984 | 4.994 | -0.598 | 0.550 |
|  | Impala [Coursing] - Zebra [Coursing] | -5.154 | 6.004 | -0.858 | 0.391 |
|  | Wildebeest [Control] - Wildebeest [Ambush] | -0.904 | 2.171 | -0.417 | 0.677 |
|  | Wildebeest [Control] - Wildebeest [Coursing] | 1.527 | 4.078 | 0.374 | 0.708 |
|  | Wildebeest [Control] - Zebra [Control] | -0.951 | 4.938 | -0.193 | 0.847 |
|  | Wildebeest [Control] - Zebra [Ambush] | 2.352 | 2.097 | 1.122 | 0.262 |
|  | Wildebeest [Control] - Zebra [Coursing] | 0.182 | 3.776 | 0.048 | 0.962 |
|  | Wildebeest [Ambush] - Wildebeest [Coursing] | 2.431 | 2.179 | 1.116 | 0.265 |
|  | Wildebeest [Ambush] - Zebra [Control] | -0.046 | 4.504 | -0.010 | 0.992 |
|  | Wildebeest [Ambush] - Zebra [Ambush] | 3.256 | 0.935 | 3.483 | 0.000 |
|  | Wildebeest [Ambush] - Zebra [Coursing] | 1.087 | 3.208 | 0.339 | 0.735 |
|  | Wildebeest [Coursing] - Zebra [Control] | -2.477 | 4.939 | -0.502 | 0.616 |
|  | Wildebeest [Coursing] - Zebra [Ambush] | 0.825 | 2.398 | 0.344 | 0.731 |
|  | Wildebeest [Coursing] - Zebra [Coursing] | -1.345 | 3.816 | -0.352 | 0.725 |
|  | Zebra [Control] - Zebra [Ambush] | 3.303 | 4.876 | 0.677 | 0.498 |
|  | Zebra [Control] - Zebra [Coursing] | 1.133 | 1.849 | 0.613 | 0.540 |
|  | Zebra [Ambush] - Zebra [Coursing] | -2.170 | 3.514 | -0.617 | 0.537 |
